# AlphaFold-predicted protein structures and small-angle X-ray scattering: insights from an extended examination of selected data in the Small-Angle Scattering Biological Data Bank

**DOI:** 10.1107/S1600576723005344

**Published:** 2023-07-20

**Authors:** Emre Brookes, Mattia Rocco, Patrice Vachette, Jill Trewhella

**Affiliations:** a Department of Chemistry and Biochemistry, University of Montana, 32 Campus Drive, Missoula, MT 59812, USA; b Proteomica e Spettrometria di Massa, IRCCS Ospedale Policlinico San Martino, Largo R. Benzi 10, Genova 16132, Italy; c Université Paris-Saclay, CEA, CNRS, Institute for Integrative Biology of the Cell (I2BC), Gif-sur-Yvette 91198, France; d School of Life and Environmental Sciences, The University of Sydney, NSW 2006, Australia; Brazilian Synchrotron Light Laboratory, Brazil

**Keywords:** small-angle X-ray scattering, SAXS, AlphaFold, ensemble modelling, structural flexibility

## Abstract

By providing predicted protein structures from nearly all known protein sequences, the artificial intelligence program AlphaFold (AF) is having a major impact on structural biology. While a stunning accuracy has been achieved for many folding units, predicted unstructured regions and the arrangement of potentially flexible linkers connecting structured domains present challenges. Focusing on single-chain structures without prosthetic groups, an earlier comparison of features derived from small-angle X-ray scattering (SAXS) data taken from the Small-Angle Scattering Biological Data Bank (SASBDB) is extended to those calculated using the corresponding AF-predicted structures. Selected SASBDB entries were carefully examined to ensure that they represented data from monodisperse protein solutions and had sufficient statistical precision and *q* resolution for reliable structural evaluation. Three examples were identified where there is clear evidence that the single AF-predicted structure cannot account for the experimental SAXS data. Instead, excellent agreement is found with ensemble models generated by allowing for flexible linkers between high-confidence predicted structured domains. A pool of representative structures was generated using a Monte Carlo method that adjusts backbone dihedral allowed angles along potentially flexible regions. A fast ensemble modelling method was employed that optimizes the fit of pair distance distribution functions [*P*(*r*) versus *r*] and intensity profiles [*I*(*q*) versus *q*] computed from the pool to their experimental counterparts. These results highlight the complementarity between AF prediction, solution SAXS and molecular dynamics/conformational sampling for structural modelling of proteins having both structured and flexible regions.

## Abstract 2

A rapid ensemble modelling method that optimizes the fit to the small-angle X-ray scattering (SAXS)-derived pair-wise distance distribution function [*P*(*r*) versus *r*] and the measured intensity profile [*I*(*q*) versus *q*] has been used to account for differences between AlphaFold-predicted and experimental SAXS profiles. By considering the confidence levels that come with the predicted structures, a conformational ensemble with potentially flexible linkers between stable folded domains can be optimized to provide representative structures.

## 1. Introduction

The neural-network-based artificial intelligence (AI) programs AlphaFold (AF) (Jumper *et al.*, 2021 ▸) and Rosetta­Fold (Baek *et al.*, 2021 ▸) have revolutionized the field of protein structure prediction from sequence. In particular, the AF consortium has produced and made publicly available a database of predicted protein structures (https://alphafold.ebi.ac.uk), first for the entire UniProt database of curated protein sequences (Tunyasuvunakool *et al.*, 2021 ▸) and more recently for the entire catalogue of genomics-derived protein sequences (UniProt Consortium, 2021 ▸). Impressively, AF-predicted structures from the CASP14 data set achieved a mean Cα r.m.s.d. accuracy of ∼1 Å (Jumper *et al.*, 2021 ▸).

The AF database has already had a major impact in structural biology, and the predicted structures are being used as templates for solving crystal structures [see *e.g.* Flower & Hurley (2021 ▸), Chai *et al.* (2021 ▸), McCoy *et al.* (2022 ▸) and Oeffner *et al.* (2022 ▸)], as an aid in the interpretation of cryoEM maps [see *e.g.* Fontana *et al.* (2022 ▸)], in assessing the accuracy of NMR structures in solution [see *e.g.* Fowler & Williamson (2022 ▸)] and to infer structure–function relationships [see *e.g.* Ferrario *et al.* (2022 ▸), Urban & Pompon (2022 ▸), Akdel *et al.* (2022 ▸) and Heo *et al.* (2022 ▸)]. Conversely, AF structures usually have regions with low confidence or poor accuracy, and experimental information has the potential to improve these predictions (Terwilliger *et al.*, 2022 ▸).

While there are many examples of AF-predicted structures having an impressive level of accuracy, there remain several challenges. The AF prediction algorithm depends on deep learning from an extensive catalogue of structures, which nonetheless is limited by training on the set of solved protein structures in the Worldwide Protein Data Bank (https://www.wwpdb.org; wwPDB Consortium, 2019 ▸). Flexibility, however, is often a necessary aspect of protein function, and the protein universe is replete with multi-domain proteins composed of structured units with flexible linkers of variable length that limit both crystallographic and cryoEM studies. Thus, we see that there are opportunities to test and complement the AF predictions with experimental techniques, notably those that are readily available in core facilities or accessible on dedicated large infrastructures.

A recent paper (Brookes & Rocco, 2022 ▸) presented a database stemming from the first two AF releases, where for each predicted structure the calculated circular dichroism (CD) spectrum, the hydrodynamic parameters, the pair-wise atomic distance distribution function *P*(*r*) versus *r* [hereinafter indicated simply as *P*(*r*)] and ancillary information are stored (https://somo.genapp.rocks/somoaf/). On the basis of the UniProt annotations, predicted initiator sequences and post-translationally cleaved pro-peptides were removed from the structures prior to the calculations. Calculated hydrodynamic parameters were employed to show that, within a given molecular mass interval, these parameters could effectively distinguish between structures and thus could be employed for rapid tests of the predicted conformation in solution (Brookes & Rocco, 2022 ▸).

The *P*(*r*) profile, which can be determined from experiment as the indirect Fourier transform of small-angle X-ray scattering (SAXS) data (Glatter, 1977 ▸; Svergun *et al.*, 1988 ▸), is quite sensitive to the relative disposition of folded units, as nicely demonstrated in a multi-domain test protein (Koch *et al.*, 2003 ▸). As such, *P*(*r*) is ideal for evaluating AF-predicted structures containing potentially flexible linkers between structured domains, noting that the solution SAXS experiment reports on the time and ensemble average of the structures present in solution. In the previous study (Brookes & Rocco, 2022 ▸) a set of solution SAXS data was selected from the Small-Angle Scattering Biological Data Bank (SASBDB) (Valentini *et al.*, 2015 ▸; https://www.sasbdb.org) where there was a corresponding AF-predicted structure. While there were several examples of good agreement between the AF-predicted *P*(*r*) profile and that derived from the SAXS data, there were a number that showed significant differences. In this study, we have returned to consider these comparisons in the context of a careful evaluation of the SAXS profiles and their associated metadata to ensure the data were from solutions of monodisperse particles, free of aggregation and interparticle correlations, which is the fundamental requirement for reliable 3D atomistic modelling of SAXS data. The AF structures were then examined for predicted unstructured and/or low-confidence regions, which were assigned as flexible segments to obtain an ensemble of structures that gave an improved fit to the SAXS data. Our initial ensemble modelling focused on fitting in real space with *P*(*r*) as the target function. This approach facilitated rapid calculations from thousands of alternative conformations generated using the *Monomer Monte Carlo* (*MMC*) simulation tool in the *SASSIE-web* suite (Curtis *et al.*, 2012 ▸; Perkins *et al.*, 2016 ▸) (https://sassie-web.chem.utk.edu/sassie2/). Evaluation in reciprocal space (*i.e.* against the measured intensity data) was also possible in reasonable time using *CRYSOL* (Svergun *et al.*, 1995 ▸). Each resulting ensemble model was then further evaluated in reciprocal space using the more physically advanced but computationally intensive *WAXSiS* program (Chen & Hub, 2014 ▸; Knight & Hub, 2015 ▸) (https://waxsis.uni-saarland.de).

## 2. Methods

Initial data sets for analysis were selected by identifying structures present in the US-SOMO-AF database (Brookes & Rocco, 2022 ▸) with corresponding experimental SAXS intensity data deposited in the SASBDB. Intensity profiles are presented here as *I*(*q*) versus *q* [hereinafter simply indicated as *I*(*q*), where *q* = (4πsinθ)/λ with θ being half the scattering angle and λ the wavelength of the incident radiation]. Mandatory requirements included that the experimental data were collected on complete single-chain structures with no prosthetic groups and from the same organism as the corresponding AF structure (Brookes & Rocco, 2022 ▸), which limited the initial SASBDB pool to 43 entries.

*P*(*r*) is related to *I*(*q*) by a Fourier transform and, as the finite experiment measurement range for *I*(*q*) prohibits an analytical solution, *P*(*r*) is generally calculated from SAXS data using indirect methods, for example as implemented in the programs *GNOM* (Svergun, 1992 ▸) or *BayesApp* (Larsen & Pedersen, 2021 ▸). Both methods yield a *P*(*r*) profile with associated errors estimated using Monte Carlo simulations. However, different *P*(*r*)-generating methods yield very different error estimates starting from the same *I*(*q*), and the question of their reliability is an open one. In this study, the *P*(*r*) profiles calculated from SASBDB intensity curves and used for comparison with model calculations and as targets for ensemble modelling were obtained with *GNOM* as implemented in *PrimusQt/ATSAS 3.1* (Manalastas-Cantos *et al.*, 2021 ▸) and, for comparison, *BayesApp* as implemented at https://somo.chem.utk.edu/bayesapp.

For each selected AF structure with a corresponding SAXS *I*(*q*) profile, the conformational space of the AF-predicted structure obtained from the US-SOMO-AF database was explored utilizing the *Monomer Monte Carlo* (*MMC*) program of *SASSIE-web* (Curtis *et al.*, 2012 ▸; Perkins *et al.*, 2016 ▸) (https://sassie-web.chem.utk.edu/sassie2/), where the backbone di­hedral allowed angles along chosen segments of the protein are changed in sequential discrete steps. Except for the residue ranges for flexible regions and a number of trial attempts that are detailed in Table S1 in the supporting information, the default *MMC* parameters were used.

*MMC* flexible regions were selected by visual inspection of the AF structures’ low-confidence regions. *MMC* rejects structures with steric clashes, and it is recommended to run 10 000 to 50 000 trials to sample the conformational space adequately. From the *MMC* pool of accepted structures (the ‘original pool’) we selected a subset by extracting every ‘sub-selection stride’ from the *MMC*-produced multi-structure PDB file with the *mdconvert* program of *MDTraj* (Version 1.9.4; McGibbon *et al.*, 2015 ▸) utilizing the XSEDE (Towns *et al.*, 2014 ▸) allocated Jetstream2 (Hancock *et al.*, 2021 ▸) cloud-computing resource. In each case, the distribution of *R*_g_ values for this final subset of structures was compared with that of the original pool to ensure that it was representative. This representative pool will hereafter be referred to simply as ‘the pool.’

Each multi-structure PDB file was processed by the open-source hydrodynamic and SAS data analysis and simulation program *US-SOMO* (Brookes & Rocco, 2018 ▸; Revision 6730+, https://somo.aucsolutions.com/) in batch mode to compute *R*_g_, predicted *P*(*r*) profiles (normalized by the sample molecular weight and with a 1 Å bin size) and *I*(*q*) curves generated using *CRYSOL* (Svergun *et al.*, 1995 ▸). The *P*(*r*) profiles were computed on the dry structures as (Brookes *et al.*, 2013 ▸)

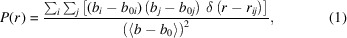

where *b_i_* and *b_j_* are the numbers of electrons of the *i* and *j* atomic groups, respectively, and the *b*_0*i*_ and *b*_0*j*_ terms account for the solvent scattering density. For SAXS, *b*_0*i*_ = 10 × (*r_i_*/*r*_wat_)^3^, where 10 is the number of electrons in a water molecule, *r_i_* is the radius of the *i*th atom and *r*_wat_ is the radius of a bulk water molecule (1.93 Å). The Kronecker delta δ(*r* − *r_ij_*) is applied to the distances *r_ij_* between the centres of atoms *i* and *j* for every bin *r*. While the contribution of the hydration layer is not considered in this implementation of the *P*(*r*) calculation, its effect is relatively minor. Tests comparing the *P*(*r*) calculated on the starting dry AF structures with those derived from a *WAXSiS*-generated *I*(*q*) profile (see below) indicate a shift of about 1 Å of the global pattern toward shorter *r* values, and some local differences mainly in the amplitudes (data not shown). Schemes utilizing explicit hydration of the starting structures are computationally intensive (and not implemented within *US-SOMO*). The approach used here allows for fast processing of thousands of structures as a preliminary screening step to generate a pool of suitable structures that are then evaluated taking into account the contribution of hydration (see below).

For this study, the *US-SOMO* batch processing protocol for generating *I*(*q*) profiles of up to a few thousand structures utilized *CRYSOL* (Version 2.8; Svergun *et al.*, 1995 ▸), with 25 for the maximum number of spherical harmonics, 18 for the order of Fibonacci’s grid, 0.335 e Å^−3^ for the solvent electron density and 0.02 e Å^−3^ for the hydration shell contrast, using the same *q* grid as the experimental one. Running on an eight-core Intel Core i7-4790 CPU/16 GB RAM workstation (Linux Ubuntu 16.04.7 LTS) for the Q16543 AF structure (*M*_w_ 44 459 Da, 378 residues) it takes ∼27 and ∼424 s for every 100 structures to compute the *P*(*r*) with 170 bins and the *I*(*q*) with a *q* grid of 1869 points, respectively. A more recent *CRYSOL* release (Version 3.2) has an option to use a different hydration scheme with dummy water beads, which should in principle be more efficient in dealing with structures presenting extended non-structured segments (Franke *et al.*, 2017 ▸). This version has now been made accessible from within *US-SOMO* and will be made available to the general user in the next planned release. As a check, we repeated all *I*(*q*) calculations with *CRYSOL 3.2* using the dummy water beads option, and a comparison of the results obtained with *CRYSOL 2.8* is presented in the supporting information (Section S1 and Tables S2–S4) with brief summary conclusions provided in the *Discussion* [Sec sec5] below.

*GNOM* *P*(*r*) curves derived from the experimental data were automatically rebinned to 1 Å steps, with errors interpolated upon loading the *GNOM* *.out file into *US-SOMO*. The *NNLS* (non-negatively constrained least-squares) utility of *US-SOMO* (Brookes *et al.*, 2016 ▸) was then used to fit the predicted *P*(*r*) and *I*(*q*) curves to their experimentally derived counterparts. *NNLS* optimization minimizes ||*Ax* − *b*||_2_ subject to *x* ≥ 0, where *A* is an *m* × *n* matrix, *x* an *n* vector and *b* an *m* vector. When the *n* columns of *A* are populated with predicted profiles and *b* with the experimental data, the algorithm, based on projections, produces a result as an *x* vector populated with zeros or positive numbers representing the fractions of the corresponding predicted profiles (Lawson & Hanson, 1995 ▸). There are no restrictions on the number of columns of *A* (number of predicted profiles) but, given a sufficiently large number of columns or solution-contributing predicted profiles that are positive linear combinations of other predicted profiles, differing collections could provide the same minimum value for ||*Ax* − *b*||_2_ on the half-space *x* ≥ 0. *NNLS* produces only one such collection. The projection-set nature of the algorithm apparently tends to provide the solution with the minimum number of predictions. No rebinning was performed on the original reference SAXS *I*(*q*) curves, notwithstanding their evident oversampling at high *q* values. While this will lead to somewhat artificially low χ^2^ values as a goodness-of-fit measure, here we are not concerned with their absolute values but only with changes between the starting structure profiles and the *NNLS*-selected composite ones. Moreover, with the logarithmic rebinning needed to reduce the oversampling effectively, important features in the error-weighted residual *I*(*q*) plots between experiment and model would be suppressed, such as oscillating features that signify differences in mean dispositions of domains.

Structures identified by *NNLS* as contributing to the real-space *P*(*r*) and to the reciprocal-space *I*(*q*) *CRYSOL*-based curves were further processed by the more computationally intensive program *WAXSiS*. This program uses a short explicit solvent molecular dynamics simulation to build a spatial envelope containing the structure and its solvation shell, while constraining the backbone atoms with a harmonic potential to ensure no conformational deviations from the input structure. The computation of the excluded-solvent scattering is based on a pure-water simulation and a SAXS *I*(*q*) curve is computed that accounts for the hydration contribution (Chen & Hub, 2014 ▸; Knight & Hub, 2015 ▸). *WAXSiS* calculations used the default options except for a thorough convergence choice and using the experimental *I*(*q*) curve to define the *q* range and interval to produce predicted *I*(*q*) curves that were also subsequently *NNLS*-fitted to the experimental *I*(*q*) curve by *US-SOMO*.

Root-mean-square (r.m.s.) average radii of gyration {[〈(*R*_g_)^2^〉]^1/2^, hereinafter indicated simply as 〈*R*_g_〉} were calculated from the computed *R*_g_ of each dry structure and from the Guinier *R*_g_ reported by *WAXSiS*, weighted by their fractions in the *NNLS* fit (PDB and *WAXSiS* 〈*R*_g_〉 values are given in Table 2). As these two values were always very close, an average between the two is quoted in Section 4 below.

Directly computing χ^2^ for the *P*(*r*) *NNLS* fits presented some issues related to the limited number of points and the reliability of their associated errors when used as weights. Further, there is merit in evaluating the model fits against the measured *I*(*q*) data. Therefore, from the *WAXSiS*-calculated *I*(*q*) of each selected *MMC* structure, a sum weighted by the respective fractions from the *P*(*r*) *NNLS* fit was produced. The resulting composite *I*(*q*) curve was then scaled against the original data, yielding χ^2^ values over the same data range and with the same number of points as those determined for the other *NNLS* fits, which then could be meaningfully compared.

Graphs were prepared using *Origin* (Version 6.0; Microcal) or *OriginLab 2019b* (https://www.originlab.com). Atomistic structure figures were prepared with *UCSF Chimera* (Version 1.15; Pettersen *et al.*, 2004 ▸) using the ‘supersmooth’ ribbon representation, and superpositions were done on all atoms in the selected residues using *Chimera*’s *MatchMaker*. Figures were assembled using *PaintShopPro* (Version 5.3; JASC Software, now Corel, https://www.paintshoppro.com).

## 3. Selection of AF-predicted structures with corresponding experimental SAXS data and evaluation of SAXS data quality

**Figure 1:**
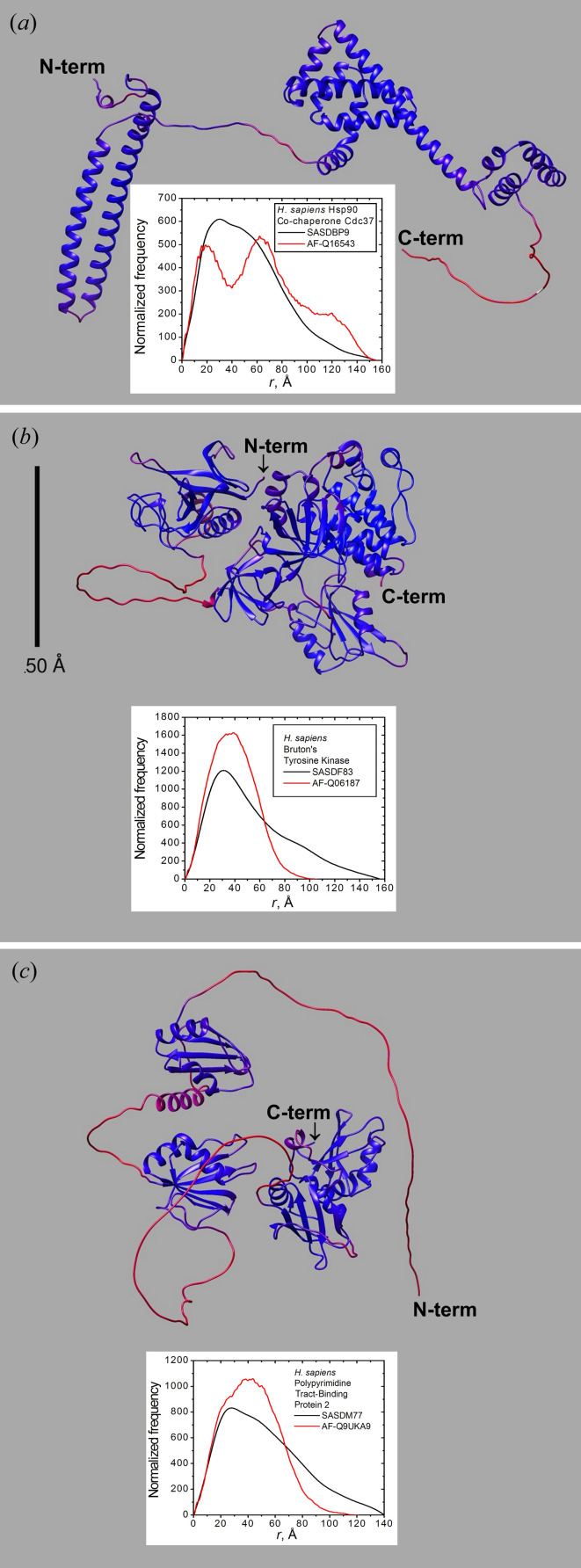
Ribbon representations of each selected AF-predicted structure, colour-coded from red to blue according to an increasing confidence level. (*a*) Q16543 (*Homo sapiens* Hsp90 co-chaperone Cdc37, residues 1–378). (*b*) Q06187 (*H. sapiens* Bruton’s tyrosine kinase, mature protein residues 2–659). (*c*) Q9UKA9 (*H. sapiens* polypyrimidine tract-binding protein 2, residues 1–531). The insets show their *P*(*r*) profiles calculated from the dry structures, and SAXS-derived profiles as retrieved from SASBDB (red and black lines, respectively). The scale bar shown in (*b*) also applies to the other panels.

An initial qualitative survey of SAXS data sets with corresponding AF-predicted structures meeting the requirements detailed above (in Section 2) revealed three candidates where the AF-predicted and SAXS-derived *P*(*r*) values are significantly different: AF-Q16543, AF-Q06187 and AF-Q9UKA9, with corresponding SAXS data SASDBP9 (Bunney *et al.*, 2018 ▸), SASDF83 (Duarte *et al.*, 2020 ▸) and SASDM77 (Simpson *et al.*, 2004 ▸), respectively (Fig. 1 ▸). In each case there were extended low-confidence regions in the AF-predicted structure that might signify flexibility. Having identified these three potential candidate structures that appear to require modification to represent the solution state properly, we proceeded to assess the quality of the SAXS data and their suitability for modelling.

Solution SAXS data from proteins with substantial flexibility present several challenges. First, they are more susceptible to small degrees of aggregation compared with folded structures, so it is highly desirable to work with data collected using inline size-exclusion chromatography (SEC–SAXS) when available. Second, the selection of *d*_max_ for a *P*(*r*) transformation is challenging, as the *P*(*r*) from an ensemble of structures with a range of *d*_max_ values generally yields a *P*(*r*) that approaches *d*_max_ with a long, very low intensity tail with large errors that nonetheless can have a significant effect on *R*_g_ calculated from the second moment of *P*(*r*). Third, the determination of the Porod volume from the scattering invariant assumes an object of uniform scattering density and a sharp interface with the solvent. While this is an arguably valid approximation in the case of a compact fully structured protein, this is hardly relevant in the case of a flexible structure.

For each of the candidate SAXS data sets, the SAXS-derived molecular mass (*M* ^expt^) agrees reasonably with the mass calculated from the chemical composition (*M* ^calc^) (Table 1 ▸), although we note that quite different methods were used for determining *M* ^expt^ (see the footnotes to Table 1 ▸). Inspection of the *P*(*r*) transforms present in SASBDB for each of these data sets revealed that the selected *d*_max_ did not yield a *P*(*r*) profile with the expected gradual approach to a near-horizontal tangent at *d*_max_; rather there was a sharp cut-off that was most severe for SASDM77. We therefore recalculated the *P*(*r*) transforms using *GNOM* with a standardized approach for *d*_max_ selection: *d*_max_ was selected such that release of the constraint *P*(*r*) = 0 at *d*_max_ did not result in a significant increase in *P*(*r*) intensity at long *r*. The resulting *P*(*r*) profiles all showed the expected shape at long *r*, but with large errors as *r* approaches *d*_max_. *P*(*r*) profiles obtained using *BayesApp* resulted in profiles that had similar *R*_g_ values, but with *d*_max_ values that were shorter by 8–20 Å. Thus, there is a degree of uncertainty in the selection of *d*_max_ for these structures. On average, Guinier-derived *R*_g_ values were ∼2 Å smaller than those derived from *P*(*r*), except for SASDM77 where the difference was ∼4 Å, but here the error in the *P*(*r*) *R*_g_ was more than four times that for the other data sets. The *P*(*r*) fits were all acceptable, as estimated by the *GNOM* total quality estimate values (0.72–0.79). The χ^2^ values for the *P*(*r*) fits were in the range 1.03–1.15 with acceptable *CorMAP* *P* values (Franke *et al.*, 2015 ▸), except for SASDF83 (χ^2^ 1.35, *P* value 0.0003) (Table 1 ▸). The error-weighted residual difference plot for the *P*(*r*) fit for SASDF83 was nevertheless flat and featureless, with deviations predominantly in the range ±3. Alternative *d*_max_ values resulted in worse fitting parameters for this data set and it was a very narrow *q* region that was responsible for the low *P* value (0.134–0.139 Å^−1^). We thus conclude that all our *GNOM*-derived *P*(*r*) fits to experiment are acceptable.

Finally, to give an accurate characterization of a structure of maximum dimension *d*_max_, one must adequately sample *I*(*q*) in the Guinier regime and *q*_min_ should be < π/*d*_max_. The experimental *q*_min_ values for SASDBP9 and SASDF83 are 2.48 × 10^−3^ and 8.16 × 10^−3^ Å^−1^, respectively. There are 95 and 64 data points in their Guinier regions, respectively, noting that for SASDBP9 the first 25 data points are excluded from Guinier analysis due to an upturn indicative of parasitic scattering or some large particle contaminant that is sufficiently low level that it does not impact the SAXS-derived molecular mass. For SASDM77, *q*_min_ is only 1.41 × 10^−2^ Å^−1^ with just 17 data points in the Guinier region. Thus, while SASDBP9 and SASDF83 meet the minimal requirements for characterizing structures as large as 350–400 Å, the limit for SASDM77 is 220 Å, much closer to the experimentally derived *P*(*r*) *d*_max_ value range (170–176 Å).

In conclusion, while the SAXS data we identified as indicating that the AF-predicted structures required some modification to represent the solution conformations were not collected in the preferred SEC–SAXS mode, we can conclude that these batch-mode-acquired data are suitable for evaluating atomistic modelling as assessed by the data quality parameters in the 2017 guidelines for biomolecular small-angle scattering (Trewhella *et al.*, 2017 ▸).

## 4. Modelling the SAXS data

Briefly summarized, our overall approach to modelling began by first quantitatively assessing the AF structure predicted *I*(*q*) and *P*(*r*) profiles without modification against their respective experimental SAXS profiles; χ^2^ values for each AF structure were calculated using *WAXSiS*-generated *I*(*q*) profiles scaled to experiment, and Guinier and *P*(*r*)-derived structural parameters were compared. For these comparisons, and for all modelling with *P*(*r*) as the target function, the experimentally derived *P*(*r*) recalculated with the standardized approach for *d*_max_ selection target was used. Considering the observed differences plus the low-confidence predicted regions for the AF structures, the conformational space to be explored in developing an ensemble model was expanded by employing *MMC* with potentially flexible sequence segments to generate a pool of potential structures (‘the original pool’), from which a representative subset (‘the pool’) was selected. Ensemble modelling was first performed using *NNLS* with this pool of structures to optimize the fit to the experimentally derived *P*(*r*), without and with errors generated in the indirect Fourier transform. To evaluate the resulting conformational ensembles in reciprocal space, the ensemble *I*(*q*) profile was calculated by summing *WAXSiS*-generated *I*(*q*) profiles for the individual conformations in each ensemble, weighted by the *NNLS*-reported fraction. To compare results obtained by optimizing the fit to *P*(*r*) with those obtained by directly fitting in reciprocal space against the measured *I*(*q*), *NNLS* optimizations were also performed starting with the same *MMC* pool of potential structures as for the *P*(*r*) fitting but using *CRYSOL 2.8*-generated individual predicted *I*(*q*) profiles. Finally, *WAXSiS* was used to calculate *I*(*q*) profiles for all conformations present in the ensemble fits to *P*(*r*) or *I*(*q*), and these were subjected to *NNLS* optimization against the experimental *I*(*q*) profile.

### 4.1. AF-Q16543 predicted structure and SASDBP9 data

**Figure 2:**
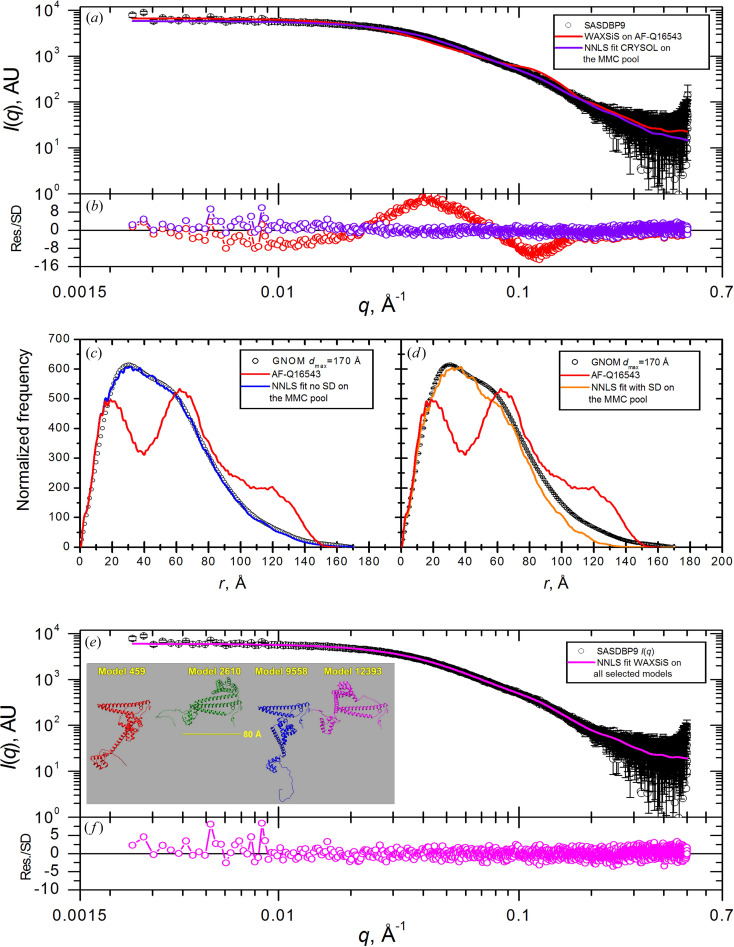
(*a*) *I*(*q*) versus *q* for SASDBP9 (black symbols with standard error bars) overlaid with the fit of the AF-Q16543 *WAXSiS*-calculated SAXS profile (red line) and with the *NNLS* ensemble fit model generated from the *CRYSOL 2.8*-calculated SAXS profiles of the *MMC* pool structures (violet line). (*b*) Error-weighted residual plots for the fits shown in panel (*a*). (*c*) and (*d*) *GNOM*-derived *P*(*r*) profiles (black symbols without/with standard error bars) overlaid with that from the AF-Q16543 prediction (red lines) and with the *NNLS* ensemble fit from the *P*(*r*) calculated on the *MMC* pool without and with error weighting, respectively (blue and orange lines). (*e*) *I*(*q*) versus *q* for SASDBP9 (black symbols with standard error bars) overlaid with the *NNLS* ensemble fit model calculated using the *WAXSiS*-generated *I*(*q*) versus *q* profiles of all *NNLS*-selected structures from the *CRYSOL 2.8* and *P*(*r*) fits (magenta line). In the inset, four representative structures selected with a significant percentage by at least two of the four *NNLS* fits are shown, after superposition on the 1–120 N-terminal residues [see Table 2 ▸(*a*) for the full fitting results]. (*f*) Error-weighted residual plot for the fit shown in panel (*e*).

The AF-Q16543 structure [Fig. 1 ▸(*a*)] is composed of two folded domains connected by an unstructured linker spanning residues 121–139, with an AF average prediction confidence level (APCL) of 66 ± 14%. There is also a long unstructured C-terminal tail (residues 343–378) with an APCL of 35 ± 10%. The *P*(*r*) calculated for the AF structure differs significantly from that deposited in the SASBDB for SASDBP9 [see Fig. 1 ▸(*a*), inset]. The AF-Q16543 *P*(*r*) has a double peak and a shoulder, indicative of multiple folded domains with dispositions that are, on average, relatively constrained. The SAXS-derived *P*(*r*) has a single peak with a shoulder and an extended tail approaching a *d*_max_ value that is similar to the AF-calculated value, suggesting a broad distribution of compact to highly extended structures, weighted towards the more compact ones, consistent with a flexible linker in solution and a potentially flexible C-terminal region. The high χ^2^ value (19.044) [Table 2 ▸(*a*)] calculated between the computed *I*(*q*) for the AF structure and the experimental *I*(*q*) after scaling [Fig. 2 ▸(*a*)] indicates that there are substantial differences between the AF-predicted structure and the solution state. The large oscillations in the error-weighted residual plot for the experimental versus predicted *I*(*q*) [Fig. 2 ▸(*b*)] (−12 to 16) indicate that the differences are highly significant.

#### 4.1.1. Ensemble modelling with *P*(*r*) as the target function

The *MMC* protocol was used to generate a pool of potential solution conformations for AF-Q16543 by allowing dihedral angle variations for sequence segments 121–139 and 343–378 (the run summary is given in Table S1). The original pool included 15 661 structures, of which 1740 representative ones (one in nine) formed the pool input to the *NNLS* tool to find the best fit to the experimentally derived *P*(*r*). Visual inspection of the *P*(*r*) fit without errors [Fig. 2 ▸(*c*)] shows excellent qualitative agreement. The resulting χ^2^ value of 1.399 [Table 2 ▸(*a*)] for the composite *WAXSiS*-calculated *I*(*q*) fit to the experimental data is more than an order of magnitude improvement compared with the unmodified structure. Furthermore, the 〈*R*_g_〉 of 41.6 Å is in excellent agreement with the SAXS-derived values [Tables 1 ▸ and 2 ▸(*a*)]. The *P*(*r*) maximum *d*_max_ value is within the uncertainty of the experimental *P*(*r*) *d*_max_, with the population weight of the structure having the longest *d*_max_ being 14%. The considerably shorter 〈*d*_max_〉 compared with the experimental *P*(*r*) *d*_max_ reflects the spread in *d*_max_ values among the selected structures. In contrast, the *NNLS* fit employing error weighting [Fig. 2 ▸(*d*)] clearly underused the contributions of structures having *d*_max_ values in the long-*r* range, where the experimentally derived *P*(*r*) has the largest errors. Notably, the χ^2^ value was significantly higher (2.065) and the 〈*R*_g_〉 and maximum *d*_max_ values were all lower than the experimentally derived ones, with an even shorter 〈*d*_max_〉 [Table 2 ▸(*a*)]. These results bring into question the utility and/or accuracy of the *P*(*r*)-associated errors in this protocol.

**Figure 3:**
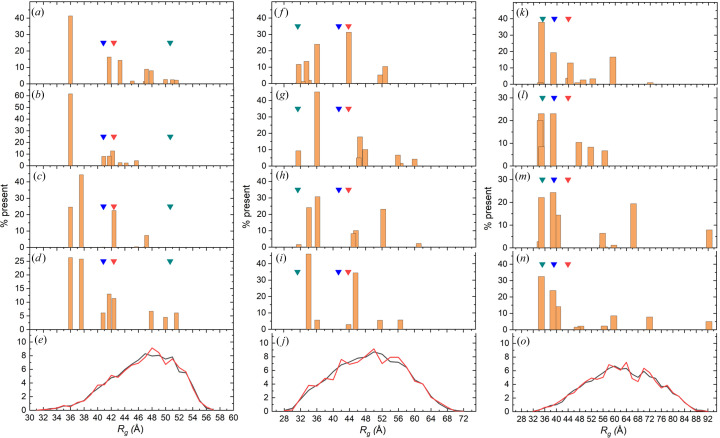
Histogram plots of the *R*_g_ values and their percent contribution for the individual structures selected by the *NNLS* fits for (*a*)–(*d*) the SASDBP9/Q16543, (*f*)–(*i*) the SASDF83/Q06187 and (*k*)–(*n*) the SASDM77/Q9UKA9 systems, respectively. (*a*), (*f*) and (*k*) *P*(*r*) *NNLS* fits without error weighting. (*b*), (*g*) and (*l*) *P*(*r*) *NNLS* fits with error weighting. (*c*), (*h*) and (*m*) *CRYSOL* *NNLS* fits. (*d*), (*i*) and (*n*) *WAXSiS* *NNLS* fits on the structures selected by all the other methods. In all histogram panels, the inverted blue and red triangles indicate the *R*_g_ values derived from Guinier and *GNOM* *P*(*r*) analyses of the experimental data, respectively, while the dark cyan inverted triangles represent the *R*_g_ of the starting AF structures. (*e*), (*j*) and (*o*) Distributions of the *R*_g_ values for the structures in the original *MMC* pool (solid black lines) and for the sub-selected *MMC* pool (solid red lines) used for the *NNLS* fitting. (*e*) Q16543, (*j*) Q06187 and (*o*) Q9UKA9.

Histograms of the percent contribution to the 〈*R*_g_〉 of individual structures in the *P*(*r*) *NNLS* fits [Figs. 3 ▸(*a*) and 3 ▸(*b*)] show that a single structure with an *R*_g_ of 36 Å accounts for ∼41 and ∼62% of the ensemble population without and with error weighting, respectively (see also Table S2). As expected from the above analysis, the histogram for *R*_g_ values obtained without error weighting is skewed towards higher values [Fig. 3 ▸(*a*)]. The two experimentally derived Guinier and *P*(*r*) *R*_g_ values are near a cluster of contributing structures with intermediate *R*_g_ values, while that for the AF structure is near a small cluster of the most extended ones, albeit with low percentage contributions, that are altogether missing from the ensemble obtained with error weighting. Inspection of the individual *P*(*r*) profiles of the contributing structures and their percent contributions for the *NNLS* fits without and with error weighting [Figs. S1(*a*) and S1(*b*), respectively] reveals that the *P*(*r*) profiles can be clustered into five and four categories, respectively. The *P*(*r*) shape clustering is supported by a similar clustering observed for the ribbon representations of each structure, after superposition on the N-terminal 25–110 sequence [Figs. S1(*c*) and S1(*d*)].

#### 4.1.2. Ensemble modelling with *I*(*q*) as the target function

The ensemble model obtained using *NNLS* with *CRYSOL*-calculated *I*(*q*) profiles for the same *MMC* pool of structures as for the *P*(*r*) fits yielded an *I*(*q*) profile fit [Figs. 2 ▸(*a*) and 2 ▸(*b*)] whose quality is comparable to the *P*(*r*) fit without error weighting, though with a slightly higher χ^2^ value [1.602, Table 2 ▸(*a*)]. The 〈*R*_g_〉 value was ∼6% lower than the experimentally derived values, and the maximum *d*_max_ value (23% contribution) was ∼9% higher than that of the *GNOM*-derived *P*(*r*), with a 〈*d*_max_〉 in between those of the *P*(*r*) *NNLS* fits [Tables 1 ▸ and 2 ▸(*a*)]. The corresponding histogram of the *R*_g_ values [Fig. 3 ▸(*c*)] shows a similar preference for more compact structures to that observed for the conformational ensemble obtained from the *P*(*r*) fit with error weighting. Three structures having *R*_g_ values in the range 36–43 Å account for more than ∼92% of the structures present, with one of them (contributing ∼22%) being close to the experimentally derived *R*_g_ values.

Finally, all the structures selected by the *NNLS* fits with either *P*(*r*) or *I*(*q*) as the target, plus the starting AF-predicted structure (a total of 22 structures), were taken as a set and calculated *WAXSiS* *I*(*q*) profiles were used in an *NNLS* fit to the SASDBP9 *I*(*q*) [Figs. 2 ▸(*e*) and 2 ▸(*f*)]. This calculation yielded the lowest χ^2^ value [1.228, Table 2 ▸(*a*)], and the 〈*d*_max_〉 and maximum *d*_max_ values are essentially the same as those found by the *NNLS* fit with *CRYSOL*-generated *I*(*q*) profiles with the larger *MMC* pool, while the 〈*R*_g_〉 values are closer to those obtained by the *NNLS* fits of the *P*(*r*) without error weighting. The corresponding *R*_g_ distribution histogram [Fig. 3 ▸(*d*)] shows two major clusters of *R*_g_ values in the ranges 36–38 and 40–43 Å, accounting for ∼49 and ∼36% of the structures, respectively, the latter encompassing experimentally derived *R*_g_ values (see Table 1 ▸). In partial contrast with the results obtained using *CRYSOL*, two more elongated structures were selected for a ∼15% contribution. Moreover, the starting AF-predicted structure, which was not selected by either the two *P*(*r*)-based fits or the *CRYSOL* fit, contributes 4.5% to the *WAXSiS*-based *NNLS* fit (Table S2).

For completeness, overlays of the SASDBP9 experimental profile with the *CRYSOL*- and *WAXSiS*-generated individual *I*(*q*) profiles of the selected structures, with their percent contributions, are shown in Figs. S2(*a*) and S2(*b*), respectively. They are accompanied by ribbon representations of the corresponding structures aligned and grouped according to the *P*(*r*)-derived classes [Fig. S2(*c*)], with a lone extra single structure that contributes ∼22 and ∼13% to the *CRYSOL* and *WAXSiS* *NNLS* fits, respectively.

Finally, the four structures with the highest percent contributions from all the *NNLS* fits [inset in Fig. 2 ▸(*e*)] all have *R*_g_ values in the range 36–42 Å, corresponding to structures in the *MMC* sets at the lower end of the *R*_g_ distribution, as shown in Fig. 3 ▸(*e*), which also highlights the good correspondence between the original entire *MMC* pool and the pool employed for the *NNLS* fits. Overall, our analysis indicates that this protein adopts significantly more compact conformations than does the starting AF-Q16543 structure.

### 4.2. AF-predicted structure Q06187 and SASDF83 data

**Figure 4:**
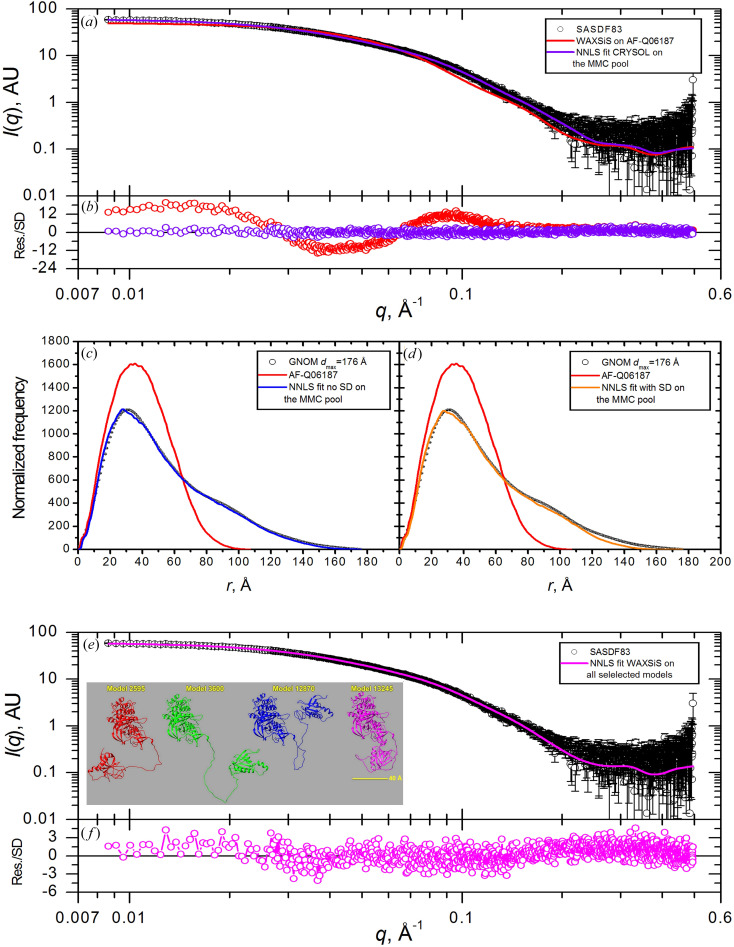
(*a*) *I*(*q*) versus *q* SAXS data taken from SASDF83 (black symbols with standard error bars) overlaid with the fit of the AF-Q06187 *WAXSiS*-calculated SAXS profile (red line) and with the *NNLS* ensemble fit model generated from the *CRYSOL 2.8*-calculated SAXS profiles on the *MMC* pool structures (violet line). (*b*) Error-weighted residual plots for the fits shown in panel (*a*). (*c*) and (*d*) *GNOM*-derived *P*(*r*) profiles from SAXS data (black symbols without/with standard error bars) overlaid with that from the AF-Q06187 prediction (red lines) and with the *NNLS* ensemble fits from the *P*(*r*) calculated on the *MMC* pool structures without and with error weighting, respectively (blue and orange lines). (*e*) *I*(*q*) versus *q* SAXS data taken from SASDF83 (black symbols with standard error bars) overlaid with the *NNLS* ensemble fit model calculated using the *WAXSiS*-generated *I*(*q*) versus *q* profiles of all *NNLS*-selected structures from the *CRYSOL 2.8* and *P*(*r*) fits (magenta line). In the inset, four representative structures selected with a significant percentage by at least two of the four *NNLS* fits are shown, after superposition on the 213–659 C-terminal residues [see Table 2 ▸(*b*) for the full fitting results]. (*f*) Error-weighted residual plot for the fit shown in panel (*e*).

The structure of AF-Q06187 [Fig. 1 ▸(*b*)] comprises an N-terminal folded domain (residues 1–169) connected by an essentially unstructured linker (residues 170–210, APCL 36 ± 5%) to a larger folded C-terminal domain (residues 211–659). Parts of both domains appear close in space, although no real contact interface is observed. The experimentally derived *P*(*r*) retrieved from the SASDF83 entry is significantly more extended (by ∼60 to 70 Å) than that calculated for the structure [see inset in Fig. 1 ▸(*b*)]. As found for the AF-Q16543/SASDBP9 case, its *WAXSiS*-generated *I*(*q*) profile gives a very poor fit to the experimental curve [Fig. 4 ▸(*a*); χ^2^ = 31.25, Table 2 ▸(*b*)] and an oscillating error-weighted residual plot (−14 to 18) [Fig. 4 ▸(*b*)]. These observations strongly suggest that the sequence segment 170–210 could be a flexible linker and the two domains are, on average, more separated in solution than in the AF-predicted structure.

#### 4.2.1. Ensemble modelling with *P*(*r*) as the target function

A total of 14 582 accepted conformations were generated using the *MMC* protocol by allowing dihedral angle variations in the 170–210 linker, from which 972 were selected (one every 15; run summary provided in Table S1) as the pool for input to the *NNLS* tool to find the optimal fit to the SASDF83 *GNOM*-derived *P*(*r*). Calculations were performed without and with error weighting [Figs. 4 ▸(*c*) and 4 ▸(*d*), respectively]. As for AF-Q16543, visual inspection indicates that the *NNLS* fits yield improved agreement, with error weighting resulting in a worse fit, as indicated by the χ^2^ values for the corresponding *WAXSiS*-based composite *I*(*q*) fit to the experimental data [1.997 and 2.716, respectively, Table 2 ▸(*b*)]. In both cases, however, there is still more than an order of magnitude improvement over the unmodified structure and the 〈*R*_g_〉 values are in reasonable agreement with the Guinier and *P*(*r*)-derived experimental *R*_g_ values [Tables 1 ▸ and 2 ▸(*b*)]. Further, the *P*(*r*)-derived longest *d*_max_ values show deviations from experimental values [Tables 1 ▸ and 2 ▸(*b*)] on a similar scale to what was observed for AF-Q16543, but the 〈*d*_max_〉 values are considerably shorter than the experimental *P*(*r*) *d*_max_, suggesting a clustering of selected structures towards more compact ones within the pool. Indeed, histograms of the percent contribution to the 〈*R*_g_〉 of individual structures in the *P*(*r*) *NNLS* fits [Figs. 3 ▸(*f*) and 3 ▸(*g*)] show a cluster with *R*_g_ values of 32–36 Å (together accounting for 53 and 55% of the contribution), close to that of the starting AF structure, with one structure being heavily selected by both fits with or without errors (see also Table S3). A single structure with an *R*_g_ value practically identical to that from the *GNOM*-derived *P*(*r*) was selected only by the *NNLS* fit without errors and with a 31% contribution. Notwithstanding the visually poorer fit at longer *r* values for the *NNLS* *P*(*r*) fit with errors, it selected structures with larger *R*_g_ values, although with relatively smaller percent contributions than for calculations without error weighting.

The individual *P*(*r*) contributions and global views of the ensembles of structures selected by the two *NNLS* fits (Fig. S3) show a similar *P*(*r*) clustering between the two calculations, with three clear classes, one of which can be split into two by the different orientation of the N-terminal domain that is absent from the *P*(*r*) *NNLS* fit with error weighting.

#### 4.2.2. Ensemble modelling with *I*(*q*) as the target function

The experimental *I*(*q*) for SASDF83 includes 1284 data points with a non-uniform *q* spacing (Δ*q*): Δ*q* is 4.696 × 10^−4^ Å^−1^ for *q* in the range [0.8164, 2.648] × 10^−2^ Å^−1^, and then Δ*q* has variable steps in the range 2.3 × 10^−6^ to 4.696 × 10^−4^ Å^−1^ up to *q* = 0.14398 Å^−1^, followed by a uniform Δ*q* = 4.713 × 10^−4^ Å^−1^ to *q*_max_ = 0.49462 Å^−1^. The *US-SOMO* *CRYSOL* implementation generated *I*(*q*) profiles for each structure from the *MMC* pool using a fixed grid spacing of 4.696 × 10^−4^ Å^−1^, yielding 1038 *I*(*q*) points that were interpolated to match the 1284 points of the experimental data for the *NNLS* fitting procedure with SASDF83 *I*(*q*) as the target. The resulting ensemble model gives a significantly improved fit to the experimental data [Figs. 4 ▸(*a*) and 4 ▸(*b*)] with a χ^2^ of 1.673 [Table 2 ▸(*b*)]. The 〈*R*_g_〉 value is, within experimental error, equal to the Guinier-derived value and lies between those of the *P*(*r*) *NNLS* fits [Tables 1 ▸ and 2 ▸(*b*)]. The histogram of the *R*_g_ distribution for the selected structures [Fig. 3 ▸(*h*)] is similar to that obtained from the *P*(*r*) *NNLS* fit with error weighting, with the cluster at 32–36 Å accounting for ∼60% of the contribution. The 〈*d*_max_〉 is similar to the *P*(*r*) *NNLS* values and the maximum *d*_max_ is close, within the uncertainties, to the *GNOM*-derived value from experimental data, with the most extended structure being present at a very low (2%) contribution [Tables 1 ▸ and 2 ▸(*b*)].

The *WAXSiS*-generated *I*(*q*) profiles from all the structures selected for any of the *NNLS* fits plus the starting AF-predicted structure (for a total of 21 structures) were then used as input for an *NNLS* fit against the SASDF83 *I*(*q*) profile [Figs. 4 ▸(*e*) and 4 ▸(*f*)]. The χ^2^ value of 1.763 for this fit is just slightly worse than that of the fit using only the *CRYSOL*-generated *I*(*q*) profiles from the *MMC* pool. The 〈*R*_g_〉 is again almost identical to the Guinier-derived value, the 〈*d*_max_〉 is similar to that found for all other fits and the structure with the longest *d*_max_ (6% contribution) is very similar to the experimentally derived value [Tables 1 ▸ and 2 ▸(*b*)]. The histogram of *R*_g_ values for the selected structures [Fig. 3 ▸(*i*)] shows two major peaks, at 34 and 46 Å, with 46 and 34% contributions, respectively, reflecting significant populations of more extended conformations compared with the AF starting structure (which was not selected; see also Table S3).

The SASDF83 experimental profile is overlaid with the *CRYSOL*- and *WAXSiS*-generated individual *I*(*q*) profiles of the selected structures with their percent contributions in Figs. S4(*a*) and S4(*b*), respectively, accompanied by the corresponding structures as ribbon representations grouped according to the *P*(*r*)-derived classes [Fig. S4(*c*), same orientations as in Figs. S3(*c*) and S3(*d*)]. Here an additional compact structure, with a similar *R*_g_ value to the second compact class present in Fig. S3(*c*) but a different orientation of the N-terminal domain, is heavily selected by the *CRYSOL* *NNLS* fit (∼33%) and is also present in the *WAXSiS* fit (∼6%). The intermediate class having a lone structure in Fig. S3(*c*) is more populated in the *I*(*q*) fits [Fig. S4(*c*)], as is the more extended structural class.

The four structures with the highest percent contributions from all *NNLS* fits are shown as ribbon representations after superposition on the C-terminal 218–659 sequence [Fig. 4 ▸(*e*), inset]. The highest proportion of *R*_g_ values are all in the lower half of the *MMC*-generated structure *R*_g_ distribution (<48 Å), with smaller percent contributions in the upper range (48–62 Å), as shown in Fig. 3 ▸(*j*), which again highlights the good correspondence between the original entire *MMC* pool and the pool employed for the *NNLS* fits. Overall, our analyses indicate that this protein assumes a range of conformations in solution that on average are significantly more extended than the starting AF-predicted structure.

### 4.3. AF-Q9UKA9 predicted structure and SASDM77 data

**Figure 5:**
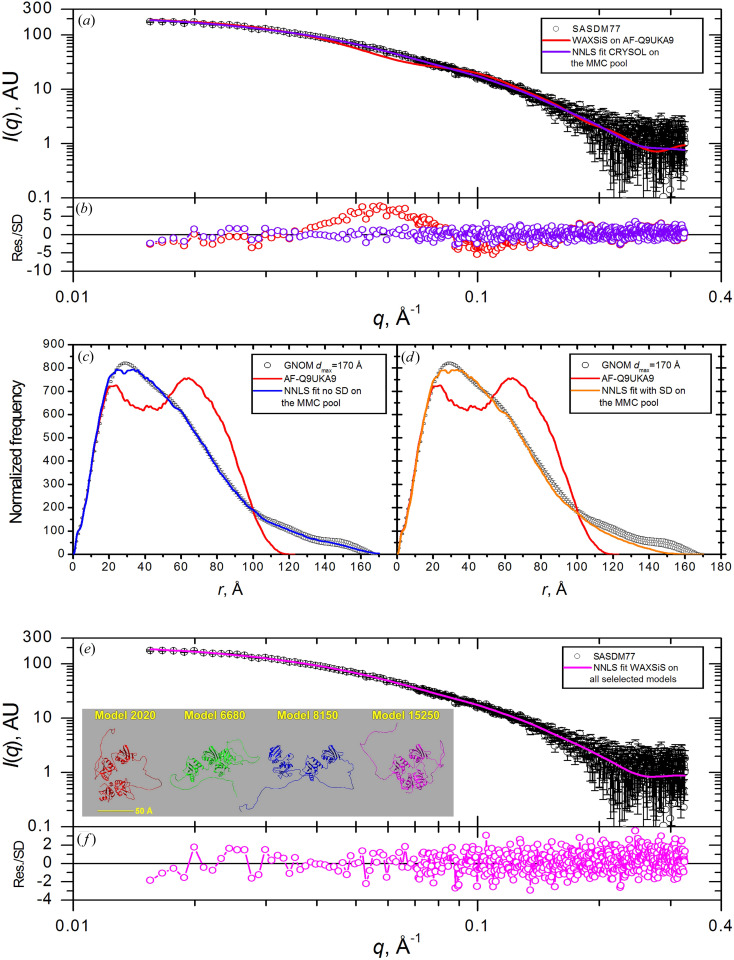
(*a*) *I*(*q*) versus *q* SAXS data taken from SASDM77 (black symbols with standard error bars) overlaid with the fit of the AF-Q9UKA9 *WAXSiS*-calculated SAXS profile (red line) and with the *NNLS* ensemble fit model generated from the *CRYSOL 2.8*-calculated SAXS profiles on the *MMC* pool structures (violet line). (*b*) Error-weighted residual plots for the fits shown in panel (*a*). (*c*) and (*d*) *GNOM*-derived *P*(*r*) profiles from SAXS data (black symbols with standard error bars) overlaid with that from the AF-Q9UKA9 prediction (red lines) and with the *NNLS* ensemble fits from *P*(*r*) calculated on the *MMC* pool structures without and with error weighting, respectively (blue and orange lines). (*e*) *I*(*q*) versus *q* SAXS data taken from SASDM77 (black symbols with standard error bars) overlaid with the *NNLS* ensemble fit model calculated using the *WAXSiS*-generated *I*(*q*) versus *q* profiles of all *NNLS*-selected structures from the *CRYSOL 2.8* and *P*(*r*) fits (magenta line). In the inset, four representative structures selected with a significant percentage by at least two of the four *NNLS* fits are shown, after superposition on the 63–270 N-terminal residues [see Table 2 ▸(*c*) for the full fitting results]. (*f*) Error-weighted residual plot for the fit shown in panel (*e*).

The structure of AF-Q9UKA9 [Fig. 1 ▸(*c*)] comprises two small N-terminal folded domains (residues 58–159 and 177–272) connected by a relatively short predicted unstructured segment (APCL 35 ± 4%) and linked by a much longer predicted unstructured segment (residues 273–336, APCL 42 ± 13%) to a C-terminal domain (residues 337–531) where two subdomains appear to have a defined interface between them. At the N-terminal there is also a long predicted unstructured segment (residues 1–57, APCL 36 ± 4%). As for the AF-Q06187 case, the experimentally derived *P*(*r*) retrieved from the SASDM77 entry is more extended than that calculated for the structure [see inset in Fig. 1 ▸(*c*)], albeit to a lesser extent (by ∼20 Å). From Fig. 5 ▸(*a*) it is evident that the *WAXSiS*-generated *I*(*q*) fits poorly, although with a relatively lower χ^2^ value (3.674) that in part reflects the larger statistical errors in this data set compared with our other two examples. Significantly, there is a clear oscillation in the error-weighted residual plot (−5 to 8) that is most evident in the intermediate *q* range, ∼0.04 to ∼0.15 Å^−1^ [Fig. 5 ▸(*b*)]. These observations suggest that some of the potentially unstructured regions could be flexible in solution, resulting in variable spatial dispositions between the domains.

#### 4.3.1. Ensemble modelling with *P*(*r*) as the target function

A first test was conducted with *MMC* where only the N-terminal 1–57 segment was allowed to be flexible, but this resulted in very minor changes in the *P*(*r*) distribution that could not account for the observed differences from experiment (data not shown). We then included in the *MMC* run the predicted unstructured linker (residues 273–336) plus the N-terminal 1–57 segment, choosing not to add the potential additional short low-confidence sequence segment (residues 160–176) to limit the degrees of freedom in the modelling. The *MMC* run allowing the dihedral angles of the two sequence segments to vary yielded 17 284 conformations, from which 1728 conformations were selected (one in every ten; run summary provided in Table S1) for *NNLS* fitting to the SASDM77-derived *P*(*r*). Visually good *NNLS* fits were obtained, without and with error weighting [Figs. 5 ▸(*c*) and 5 ▸(*d*)]. As found for the other two examples, a better fit is obtained without error weighting, most evident in the long tail at long *r* where the *P*(*r*) errors are largest. The χ^2^ values for the fits to the measured *I*(*q*) of the *WAXSiS*-generated composite *I*(*q*) profiles for all the selected structures, weighted by their contribution, were 1.279 and 1.493, respectively [Table 2 ▸(*c*)], which are 3- and 2.5-fold improvements, respectively, compared with the starting AF-predicted structure. The 〈*R*_g_〉 for the *NNLS* fit with error weighting is very close to the Guinier-derived value, while when error weighting for the fit was omitted a close match with the larger *GNOM*-derived values was obtained [Tables 1 ▸ and 2 ▸(*c*)]. The *P*(*r*)-derived longest *d*_max_ value for the *NNLS* fit with errors was essentially the same as the *GNOM*-derived value, but 22 Å longer without error weighting [Tables 1 ▸ and 2 ▸(*c*)]. The 〈*d*_max_〉 values are smaller than the *GNOM*-derived *P*(*r*) *d*_max_, suggesting a higher proportion of more compact structures selected in the fit. Histograms of the percent contribution to the 〈*R*_g_〉 of individual structures in the *P*(*r*) *NNLS* fits [Figs. 3 ▸(*k*) and 3 ▸(*l*)] indeed show a predominant cluster of structures with small *R*_g_ values (34–40 Å) comparable to that of the starting AF structure, and very close to the Guinier-derived value, accounting for 58 and 75% of the contribution, with two being strongly selected by fits with or without error weighting. Fitting without error weighting gave a single structure contributing significantly (∼10%) to a higher *R*_g_ value (see also Table S4).

The individual *P*(*r*) contributions and global views of the ensembles of structures selected by the two *NNLS* fits are shown in Fig. S5. A broad distribution of *P*(*r*) profiles is apparent in the *NNLS* fit without errors [Fig. S5(*a*)], which is somewhat reduced with error weighting [Fig. S5(*b*)]. Four and three principal structural classes could be defined, respectively [Figs. S5(*c*) and S5(*d*)], with a larger percentage of contributing structures clustering in the low-*R*_g_ range. A few structures having a wide separation between the domains are more present in the *NNLS* fits without errors.

#### 4.3.2. Ensemble modelling with *I*(*q*) as the target function

As for SASDF83, the experimental *I*(*q*) for SASDM77 does not have a uniform Δ*q*: Δ*q* is 1.102 × 10^−3^ Å^−1^ for *q* ≤ 6.9519 × 10^−2^ Å^−1^, thereafter having a variable step (Δ*q* in the range [3.53, 4.53] × 10^−4^ Å^−1^) to *q*_max_ = 0.32532 Å^−1^. *CRYSOL* generated *I*(*q*) profiles on the *MMC* pool using a fixed grid spacing of 1.102 × 10^−3^ Å^−1^, resulting in 295 *I*(*q*) points that were interpolated to match the 644 experimental data points for the *NNLS* fitting procedure. An excellent fit [Figs. 5 ▸(*a*) and 5 ▸(*b*)] was obtained with a χ^2^ of 1.208, comparable to the value of 1.279 obtained with the *P*(*r*) *NNLS* fit without errors [Table 2 ▸(*c*)]. However, the 〈*R*_g_〉 value of 52.1 Å is significantly larger than the *R*_g_ of both the *P*(*r*) *NNLS* fits and the experimental values [Tables 1 ▸ and 2 ▸(*c*)]. While the 〈*d*_max_〉 value in this case is close to the *GNOM*-derived *d*_max_ value, the maximum *d*_max_ is also greater than the experimentally derived value (243 versus 170 Å), with a 7% contribution [Tables 1 ▸ and 2 ▸(*c*)]. These results are reflected in the *R*_g_ histogram [Fig. 3 ▸(*m*)] where, together with the cluster at *R*_g_ values smaller than or near to that of the starting AF structure or close to the Guinier-derived value, there are individual structures with quite large *R*_g_ values, up to ∼92 Å for a structure that is 7% of the ensemble population.

The *WAXSiS*-generated *I*(*q*) profiles from all the structures selected for any of the *NNLS* fits, plus the starting AF structure (a total of 24 structures), were then used as input for an *NNLS* fit against the SASDM77 *I*(*q*) profile [Figs. 5 ▸(*e*) and 5 ▸(*f*)], resulting in an excellent fit with the best χ^2^ of 1.179 among all the *NNLS* fits performed on this sample [Table 2 ▸(*c*)]. The quite high 〈*R*_g_〉 value of ∼48.4 Å, as well as the 〈*d*_max_〉 and maximum *d*_max_, with the most elongated structure contributing 4% to the ensemble, are in line with the values obtained with the *CRYSOL*/*NNLS* fit [Table 2 ▸(*c*)]. The *R*_g_ histogram [Fig. 3 ▸(*n*)] shows, however, that in this case the cluster at smaller *R*_g_ values (more extended than in the *CRYSOL* fit case, 35–45 Å) is even more predominant, accounting for 75% of the structures selected, and spanning from the AF starting structure (which was not selected; see also Table S4) to those of the *GNOM*-derived values.

The individual *I*(*q*) profiles selected in the *CRYSOL* and *WAXSiS* *NNLS* fits can be seen in Figs. S6(*a*) and S6(*b*), respectively, with the corresponding structural classes in Fig. S6(*c*) [same superposition and orientations as those in Figs. S5(*c*) and S5(*d*)]. The compact conformational cluster can be split into two, followed by two clusters of relatively and quite extended structures, respectively, and the most extended one can be set apart, also based on its very peculiar *I*(*q*) profile [pink lines in Figs. S6(*a*) and S6(*b*)].

As with the other two systems studied, the four structures with the highest percent contributions from all *NNLS* fits are shown as ribbon representations after superposition on the N-terminal 63–270 domain [Fig. 5 ▸(*e*), inset]. The highest proportion of *R*_g_ values are all in the ascending half of the *MMC*-generated pool *R*_g_ distribution (32–60 Å), with smaller percent contributions in the other half (60–92 Å), selected only by the *I*(*q*) *NNLS* fits as shown in Fig. 3 ▸(*o*), which also confirms the good correspondence between the original entire *MMC* pool and the pool employed for the *NNLS* fits.

This example is distinct from the other two in that the populations obtained by modelling with *P*(*r*) versus *I*(*q*) as the target give different results with respect to the population of extended structures present, reflected in both the 〈*R*_g_〉 and maximum *P*(*r*) *d*_max_ values. It has already been noted that the SAXS data for this example are of poorer statistical quality than the other two data sets, and it is also the case that *q*_min_ is only 0.0141 Å^−1^ (compared with 0.0025 and 0.0082 Å^−1^ for SASDBP9 and SASDF83, respectively), with far fewer data points in the Guinier region (by factors of three to five). These observations raise the question of whether the low-*q* limit and sampling frequency in the Guinier regime for SASDM77 are sufficient for a reliable characterization of the most extended structures present in the sample. For this data set, the experimental *q*_min_ is such that structures with *d*_max_ > 220 Å^−1^ would not be reliably characterized, and it would also limit the accuracy of *d*_max_ when calculating *P*(*r*). Modelling with *P*(*r*) as the target may thus artificially limit *d*_max_, while modelling with *I*(*q*) as the target would probably allow for more extended structures.

With some question as to the precise nature of the population of extended structures, our analysis nevertheless suggests that the predominant confomations for this protein are just slightly more extended than that of the starting AF structure but can experience transitions to very elongated conformations in a relatively low proportion of the total population.

## 5. Discussion

AlphaFold has been shown to provide excellent predictions for large numbers of proteins. However, proteins with flexible segments cannot be adequately represented with a single static structure. Although this could be seen as a weakness of AF, it simply reflects what is often a necessary aspect in protein function. Coupling AF predictions with experimentally derived constraints and conformational space expansion could provide the researcher with an enhanced representation of the system under study.

Indeed, for the three examples identified here where there is an AF-predicted structure and a corresponding SAXS data set, we have shown that the AF-predicted structure cannot account for the experimental data without modifications. Using the confidence level indicators provided with each AF-predicted structure, we identified potential flexible linkers connecting the higher-confidence structured domains. Using the *MMC* routine, which efficiently creates tens of thousands of plausible all-atom structures with torsion angles ϕ and ψ in the allowed regions of the Ramachandran plot, we generated a pool of conformers from which a weighted population was identified that predicted the experimental SAXS data. In two cases (SASDBP9 and SASDF83), modelling with the calculated *P*(*r*) as the target gave similar results, in terms of the range and average *R*_g_ and *d*_max_ values, to modelling with *I*(*q*) as the target. In the third case, there were strong similarities but with a difference in the population of the most extended structures present in the optimized ensemble. The result obtained when modelling *I*(*q*) included significantly more extended structures than those obtained when modelling *P*(*r*). This difference appears to be attributable to a too-large *q*_min_ and Δ*q* in the Guinier region for the SASDM77 data to characterize reliably the structures with the longest *d*_max_ values. These data were collected in 2004 on the European Molecular Biology Laboratory (EMBL) X33 beamline at the DORIS III storage ring (Hamburg, Germany) using a 1D gas detector (Blanchet *et al.*, 2012 ▸) that has since been decommissioned. Improvements in instrumentation since then deliver data of a quality and *q* range more in line with the SASDBP9 data set (collected on the P12 beamline of EMBL at the storage ring PETRA-III of the Deutsches Elektronen-Synchrotron, Hamburg, using a Pilatus 2M detector) and SASDF83 data set [collected on the BM29 beamline at the ESRF (Grenoble, France) using a Dectris Pilatus 1M 2D detector (Pernot *et al.*, 2013 ▸)], which would potentially resolve the discrepancy observed here for SASDM77 modelling with *P*(*r*) versus *I*(*q*) as the target.

Our results demonstrate that modelling with *P*(*r*) as the target can give reliable results, provided that the SAXS data meet quality metrics that ensure the data represent monodisperse proteins in solution, free of interparticle correlations, *and both* *q*_min_ and Δ*q* meet the requirements for reliable characterization of the most extended structures present. Depending on the specific system, the scattering curve *I*(*q*) may be a more sensitive reporter of a global conformational change than the *P*(*r*) profile. Conversely, the *P*(*r*) can exhibit a wealth of structural features associated with domain shapes and their arrangement within the molecule that are not evident in an apparently featureless scattering curve. Computationally, the main advantage for modelling in real space is the ease and speed compared with the much more intensive *I*(*q*) calculation, especially if methods relying on explicit water all-atom molecular dynamics to account for hydration are used, *e.g.* as in the case for *WAXSiS*. While our *P*(*r*) calculations from individual structure coordinates did not account for the scattering contribution from hydration water, this effect is relatively minor compared with the difference observed between the AF structure and experimentally derived *P*(*r*), and it also becomes less significant as the protein size increases. Nevertheless, it could account for some of the differences observed between *P*(*r*) and *I*(*q*) *NNLS* fits, as both *WAXSiS* and *CRYSOL* consider the contribution from hydration water. On the other hand, if we compare the models pre-selected by *P*(*r*)- and *I*(*q*)-based methods (Tables S2–S4) we see that, for AF-Q16543, of the eight models selected by *NNLS* on the *WAXSiS*-generated *I*(*q*), three (with a combined 47% contribution) were also picked at the dry *P*(*r*) level. For the AF-Q06187 system, the numbers were five (with a combined 65% contribution) over six, and for the AF-Q9UKA9 system the numbers were six (with a combined 70% contribution) over ten. In any case, the development of methods to account reliably for hydration water in computing the *P*(*r*) from dry structures without sacrificing its speed advantage, such as those based on the statistical distributions of water used in *US-SOMO* to compute hydrodynamic properties (Rai *et al.*, 2005 ▸), would constitute a welcome improvement.

The main challenge for modelling the real-space function lies in the fact that the experimentally derived *P*(*r*) is obtained as an indirect Fourier transform that often includes a user-selected *d*_max_ value along with assumptions that *P*(*r*) goes to zero at *r* = 0 and *d*_max_. For flexible structures, depending on the nature of the population and the measured *q*_min_, there can be significant uncertainty in *d*_max_, with *P*(*r*) exhibiting a long low-intensity tail with large errors. Using multiple methods to calculate *P*(*r*) can provide a measure of the uncertainty in *d*_max_, and here we compared *GNOM*-derived *P*(*r*) profiles obtained using a standard approach to *d*_max_ selection and a Bayesian application (*BayesApp*) without a user-selected *d*_max_. The latter method generally yielded somewhat smaller *d*_max_ values, and almost an order of magnitude smaller uncertainties. We repeated the *NNLS* analysis for SASDBP9 and found very similar results to those observed using the *GNOM*-derived *P*(*r*) (data not shown), indicating that the low-intensity large-error tail on the *P*(*r*) was not very influential.

Regardless of the modelling method used, best practice is always to assess the fit against the actual measured data, which for SAXS is the *I*(*q*) profile. In all three cases tested here, the best-fit ensembles from the *P*(*r*) modelling also gave good fits to the experimental *I*(*q*) as assessed by χ^2^ and error-weighted residual plots. While error weighting in our *P*(*r*) fitting resulted in some differences, further work would be required to understand how to account for the errors properly, as they are not true counting statistics. Indeed, we have observed differences in the magnitude of errors up to a factor of ten between different software programs computing *P*(*r*) from *I*(*q*).

The computation of the *I*(*q*) profile from a structure is also dependent, among other things, on the treatment of the hydration contribution. Several computational methods are available, and a comparison of some of the most widely used ones has been presented in a recent benchmarking study (Trewhella *et al.*, 2022 ▸). To complement our *P*(*r*)-based pre-selections we have chosen *CRYSOL*, as implemented within *US-SOMO* (Brookes & Rocco, 2018 ▸), which has proven to be fast enough to allow batch-mode computation over a few thousand structures without resorting to high-end computing facilities. The main results presented here were based on *CRYSOL 2.8*, which approximates the hydration as a layer of uniform density and uniform thickness. The calculations were redone with *CRYSOL 3.2* using the more recently implemented option for explicit representation of hydration as dummy beads (Franke *et al.*, 2017 ▸), which is in principle better suited to structures presenting extended unstructured regions. However, relatively minor differences were observed (see Section S1). Moreover, when a more advanced computational approach was employed that uses a short molecular dynamics simulation within a full solvation box, namely *WAXSiS* (Chen & Hub, 2014 ▸; Knight & Hub, 2015 ▸), very similar results were obtained using structures pre-selected by the *P*(*r*) approach and either *CRYSOL 2.8* or *CRYSOL 3.2*, or both (Section S1 and Tables S2–S4). Possibly, when a structure contains a mix of folded and unstructured regions, the differences in the hydration models are not as significant compared with the case of, for instance, an intrinsically disordered protein.

For this study, we were dependent on the chance co­incidence of there being a SAXS data set corresponding to an AF-predicted structure where significant differences were apparent between the predicted and experimentally derived *P*(*r*) functions. From the initial pool of 43 data sets, just three examples were identified where the experimental data generally satisfied the quality criteria presented in the SAS guidelines (Trewhella *et al.*, 2017 ▸). While none of these were obtained using the preferred SEC–SAXS measurement mode that increases the likelihood of the sample being monodisperse, the quality assessment done demonstrates that careful measurement in batch mode can yield reliable data. Among the criteria to evaluate the quality of SAXS data, the accuracy of SAXS-derived molecular mass values is critical and not always easy to achieve. There are multiple methods available that sometimes provide differing values that should be accounted for, but there is a strong temptation simply to accept the one that gives the best agreement with expectation. For the three data sets we analysed here, three different methods were used for the reported SAXS-derived molecular mass: calculated from *I*(0) relative to a BSA standard (Mylonas & Svergun, 2007 ▸) for SASDBP9, from Bayesian inference (Hajizadeh *et al.*, 2018 ▸) for SASDF83 and from a *DAMMIN* envelope volume (Svergun, 1999 ▸) for SASDM77. In reporting SAXS data, it is highly recommended to provide SAXS-derived *M* ^expt^ values determined using multiple methods, but importantly including from *I*(0)/*c* [where *c* is the protein concentration of the sample (mass/volume)], with scattering intensities placed on an absolute scale (Trewhella *et al.*, 2017 ▸). This method requires an accurate concentration measurement of the SAXS sample and knowledge of the partial specific volume, which can be calculated from the sequence [see Trewhella *et al.* (2022 ▸)]. The uncertainty in the concentration determination, coupled with that in the partial specific volume calculation, may be the limiting factor, but for all its shortcomings, this method for estimating *M* ^expt^ is important, particularly in the case of flexible molecules for which estimates derived from the scattering curve independent of the concentration are more problematic.

We initially considered a fourth example for this study, AF-P50891 and its corresponding SASBDB data set SASDHP4, where the predicted and experimental *P*(*r*) profiles differed significantly. However, this protein presented three well characterized N-glycosylation sites (Olson *et al.*, 2020 ▸). Using the Glycam website (https://glycam.org) we built the three high-mannose carbohydrate chains to complete the atomic description of the protein, and the differences between prediction and experiment at the *I*(*q*) profile level were substantially reduced, demonstrating the importance of accounting for post-translational modifications. However, further analysis of this system was beyond the scope of this study.

Coupling of the *MMC* methodology with *NNLS* fitting in both real and reciprocal space as presented in this work has led to interesting insights in each of the systems presented. Since automation of the entire real-space analysis and of some aspects of the reciprocal-space analysis, such as internally calculated *CRYSOL* profiles and externally generated *WAXSiS* profiles, does not seem to present major hurdles, further development with a dedicated module of the *US-SOMO* online website (https://somoweb.genapp.rocks) is planned. This additional useful tool for biomolecular SAXS would nicely complement the very important advances that AlphaFold has brought to the wider biostructural community.

## Supplementary Material

Additional discussion, tables and figures. DOI: 10.1107/S1600576723005344/uu5005sup1.pdf

## Figures and Tables

**Table 1:** Parameters derived from SAXS data

SASBDB ID, AF structure ID	*M* ^calc^ (kDa)	*M* ^expt^ (kDa)	Guinier *R*_g_ (Å)	Maximum *qR*_g_	*P*(*r*) *R*_g_ (Å)†	*d*_max_ (Å)†	*P*(*r*) fit: χ^2^ ‡	*P*(*r*) fit: *P* value‡
SASDBP9, Q16543	44	49§	40.9 ± 0.5	1.0	42.4 ± 0.3	170	1.03	0.155
SASDF83, Q06187	77	67¶	41.4 ± 0.4	1.0	43.8 ± 0.3	176	1.35	0.0003
SASDM77, Q9UKA9	57	60††	39.1 ± 0.9	1.3	43.9 ± 1.1	170	1.11	0.95

† Values are for the *GNOM*-derived *P*(*r*). Values obtained using the *BayesApp*-calculated *P*(*r*) without inputting a *d*_max_ value were for *R*_g_ 41.8, 43.5 and 45.2, and for *d*_max_ 152, 152 and 162, for SASDBP9, SASDF83 and SASDM77, respectively.

‡ χ^2^ and *P* values were calculated using the *Data Comparison* tool of *PrimusQt* (Manalastas-Cantos *et al.*, 2021 ▸).

§ From *I*(0) relative to a standard, bovine serum albumin (BSA).

¶ From Bayesian inference.

†† From *DAMMIN* envelope volume, conversion factor not specified.

**Table:** 〈*R*_g_〉 stands for the r.m.s. average radius of gyration [〈(*R*_g_)^2^〉]^1/2^ (see Section 2, *Methods*). Expt. is an abbreviation for experimental and conf. is an abbreviation for conformations.(*a*) Fits to SASDBP9 based on AF-Q16543, *NNLS* fits with flexible linkers (sequence segments 121–139 and 343–378).

Fit method	*WAXSiS*, *I*(*q*), scaled	*NNLS*, expt. *P*(*r*) target		*NNLS*, expt. *I*(*q*) target	*NNLS*, expt. *I*(*q*) target
		*MMC* pool *P*(*r*)			
Structure pool	AF-Q16543	No error weighting	Error weighting	*MMC* pool [*CRYSOL 2.8* *I*(*q*)]	All *NNLS* selected conf. [*WAXSiS* *I*(*q*)]
Fit parameters (χ^2^)	19.044	1.399†	2.065†	1.602	1.228

PDB 〈*R*_g_〉 (Å)		41.7	38.6	39.2	40.8
*WAXSiS* 〈*R*_g_〉 (Å)		41.5	38.5	39.1	39.3
*P*(*r*) 〈*d*_max_〉 (Å)		157.1	139.4	146.0	150.1
*P*(*r*) max *d*_max_ (Å)		201 (14%)	158 (8%)	185 (23%)	187 (6%)

**Table:** (*b*) Fits to SASDF83 based on AF-Q06187, *NNLS* fits with flexible linker (sequence segment 170–210).

Fit method	*WAXSiS* *I*(*q*), scaled	*NNLS*, expt. *P*(*r*) target		*NNLS*, expt. *I*(*q*) target	*NNLS*, expt. *I*(*q*) target
		*MMC* pool *P*(*r*)			
Structure pool	AF-Q06187	No error weighting	Error weighting	*MMC* pool [*CRYSOL 2.8* *I*(*q*)]	All *NNLS* selected conf. [*WAXSiS* *I*(*q*)]
Fit parameters (χ^2^)	31.250	1.997†	2.716†	1.673	1.763

PDB 〈*R*_g_〉 (Å)		40.7	42.7	42.3	41.1
*WAXSiS* 〈*R*_g_〉 (Å)		40.6	42.4	42.0	41.0
*P*(*r*) 〈*d*_max_〉 (Å)		133.1	138.7	136.4	132.1
*P*(*r*) max *d*_max_ (Å)		165 (15%)	184 (4%)	187 (2%)	178 (6%)

**Table:** (*c*) Fits to SASDM77 based on AF-Q9UKA9, *NNLS* fits with flexible linkers (sequence segments 1–54 and 273–336).

Fit method	*WAXSiS*, *I*(*q*), scaled	*NNLS*, expt. *P*(*r*) target		*NNLS*, expt. *I*(*q*) target	*NNLS*, expt. *I*(*q*) target
		*MMC* pool *P*(*r*)			
Structure pool	AF-Q9UKA9	No error weighting	Error weighting	*MMC* pool [*CRYSOL 2.8* *I*(*q*)]	All *NNLS* selected conf. [*WAXSiS* *I*(*q*)]
Fit parameters (χ^2^)	3.674	1.279†	1.493†	1.208	1.179

PDB 〈*R*_g_〉 (Å)		43.8	40.4	52.2	48.4
*WAXSiS* 〈*R*_g_〉 (Å)		43.7	40.5	52.0	48.4
*P*(*r*) 〈*d*_max_〉 (Å)		152.8	143.8	162.9	152.8
*P*(*r*) max *d*_max_ (Å)		192 (4%)	168 (10%)	243 (8%)	243 (5%)

† The χ^2^ for the *P*(*r*) *NNLS* fits were determined by computing the *I*(*q*) of each selected *MMC* structure using *WAXSiS*, and then making a weighted sum using the respective fractions from the *NNLS* fit. The resulting *I*(*q*) curve was then scaled against the original data.
