# Supplementary material for: AlphaFold-predicted protein structures and small-angle X-ray scattering: insights from an extended examination of selected data in the Small-Angle Scattering Biological Data Bank
Source: J Appl Crystallogr. 2023 Jul 20;56(Pt 4):910–26. doi: 10.1107/S1600576723005344 (PMC10405597; doi:10.1107/S1600576723005344)
Supplement: Supplementary file 1 [file j-56-00910-sup1.pdf]

JOURNAL OF
APPLIED
CRYSTALLOGRAPHY

Volume 56 (2023)

Supporting information for article:

AlphaFold predicted protein structures and small-angle X-ray scattering: insights from an extended examination of selected data in the Small-Angle Scattering Database

Emre Brookes, Mattia Rocco, Patrice Vachette and Jill Trewhella

S1. CRYSOL 3.2 Calculations

As noted in the main text, a recent CRYSOL release (version 3.2) has an option to use a different hydration scheme with dummy water beads, which in principle should be more efficient in dealing with structures presenting extended non-structured segments (Franke *et al.*, 2017). Since completing the original study, CRYSOL 3.2 has been made accessible from within US-SOMO and will be made available to the general user in the next planned release. To evaluate the potential impact of the alternate hydration scheme, we repeated all the NNLS fits using $I(q)$ profiles generated using CRYSOL 3.2 with the dummy water option.

For SASDBP9 (**Table S2**), the NNLS fit with CRYSOL 3.2-generated $I(q)$ profiles yielded the same three highest contributing structures as was obtained with CRYSOL 2.8, in two cases with comparable percentages, with some differences in the minor population structures and a slightly improved χ^2 value. The subsequent WAXSiS-based NNLS fit yielded the same results as that based on the CRYSOL 2.8-produced data. When all structures based on both $P(r)$ and both CRYSOL versions NNLS fits were included, again the NNLS returned identical results as those that included only the CRYSOL 2.8-based selected structures. Thus, in this case it appears that utilizing CRYSOL 3.2 did not provide a different pre-selection of contributing structures over CRYSOL 2.8.

The NNLS fits for SASDF83 (**Table S3**) using CRYSOL 3.2-generated $I(q)$ profiles selected the same three structures contributing significantly (>10%) as for CRYSOL 2.8, but with different percentages, and, as for the AF-Q16543/SASDBP9 set, with a slightly worse χ^2 value. In this case, the subsequent WAXSiS-based NNLS selected four out of six structures in common with those selected by the WAXSiS NNLS fit including the CRYSOL 2.8 pre-selected models, with variable percentages and practically identical χ^2. The very same result was obtained when the NNLS fit was performed with WAXSiS calculated $I(q)$ profiles from structure sets selected using all methods.

The NNLS fits with the CRYSOL 3.2-generated $I(q)$ profiles for SASDM77 (**Table S4**) selected twelve structures, five of which were in common with the ten structures selected in the CRYSOL 2.8-NNLS fit, albeit with different percentages, and again a slightly worse χ^2 value. The subsequent WAXSiS-based NNLS fit produced results close to those based on the CRYSOL 2.8 selections, in many cases with similar percentages, and with a practically identical χ^2 value. In this case, when all selected structures based on both $P(r)$ and both CRYSOL versions NNLS fits were included, the NNLS returned mixed results, and with an identical χ^2 value as the other two. As in the previous two cases, using CRYSOL 3.2 over 2.8 provided no clear distinction when all models pre-selected where then analyzed based on WAXSiS computations. If anything, the CRYSOL 3.2-based NNLS fit selected two structures with a very large d_{max} (275 and 281 Å), one of which was confirmed at a significant level (structure 12,700 in **Table S4**, ~10%) by the subsequent WAXSiS-based NNLS fits.

Table S1 MMC runs summary.

AF Accession code<br>SASBDB Accession code	Q16543<br>SASDBP9	Q06817<br>SASDF83	Q9UK19<br>SASDM77
Residue ranges for flexible regions	121-139<br>343-378	170-210	1-54<br>273-336
Number of trial attempts	20,000	20,000	20,000
Accepted structures	15,661	14,582	17,284
Sub-selection stride	9	15	10
Sub-selected structures	1,740	972	1,728
AF starting structure R_g (Å)	50.0	30.5	41.4
Lowest R_g (Å)	30.5	24.1	29.0
Highest R_g (Å)	56.1	73.3	93.8
Average accepted R_g (Å)	46.5	49.9	63.3

Table S2 Models selected by NNLS from the data produced by the various methods, including CRYSOL 3.2 with dummy waters, and their percentages, for the SASDBP9/AF-Q16543 system. The WAXSiS-based NNLS fits included the two *P*(*r*) datasets and either one of the CRYSOL version-based dataset (e.g., "w CR 2.8") or both CRYSOL versions-based datasets ("all").

Model #	*P*(*r*) no err wt	*P*(*r*) err wt	CRYSOL 2.8	CRYSOL 3.2	WAXSiS w CR 2.8	WAXSiS w CR 3.2	WAXSiS all
0					4.5	4.5	4.5
90							
459			44.3	38.4	25.8	25.8	25.8
1404				1.0			
1494		4.4	0.4	6.2			
1503		2.4					
2610	41.3	61.6	24.6	32.3	26.3	26.3	26.3
4617			0.6	1.7			
5166		2.6					
6543		12.7					
7263				0.1			
7371	1.5						
7929	14.3						
7938			0.2	2.3	6.1	6.1	6.1
8208				0.6			
9009	0.6						
9387	1.7						
9558	16.3	8.3			13.0	13.0	13.0
9783				0.9			
9801	2.3				6.1	6.1	6.1
9819	2.7						
9855	8.9						
10323	2.5						
11169				11.0			
11880			7.3				
11943	8.0				6.7	6.7	6.7
12393			22.5	5.6	11.4	11.4	11.4
15237		8.0					
Sum %	100.0	100.0	100.0	100.0	100.0	100.0	100.0
χ^2	1.399	2.065	1.602	1.521	1.228	1.228	1.228

Table S3 Models selected by NNLS from the data produced by the various methods, including CRYSOL 3.2 with dummy waters, and their percentages, for the SASDF83/AF-Q06187 system. The WAXSiS-based NNLS fits included the two *P*(*r*) datasets and either one of the CRYSOL version-based dataset (e.g., "w CR 2.8") or both CRYSOL versions-based datasets ("all").

Model #	*P*(*r*) no err wt	*P*(*r*) err wt	CRYSOL 2.8	CRYSOL 3.2	WAXSiS w CR 2.8	WAXSiS w CR 3.2	WAXSiS all
0							
945							
1350		17.8					
1440			2.2				
1770	0.2	5.0					
1785		10.1					
2595	2.0		24.1	37.6	46.0	42.8	42.8
3150				0.4			
3360	13.5						
3600			10.1	39.0	34.8	23.2	23.2
4500			23.1				
6465	0.5	9.3					
7665		1.7			5.7	2.7	2.7
7680		6.7					
7725	5.2				5.4		
7830							
11850			8.2				
11925				2.8		9.3	9.3
11940				10.7			
12180	1.2						
12465		4.2					
12510	10.4						
12810	31.3				2.5	14.4	14.4
12870	24.0	45.2					
13245	11.7		1.6		5.6	7.7	7.7
13845			30.7	9.5			
Sum %	100.0	100.0	100.0	100.0	100.0	100.0	100.0
χ^2	1.997	2.716	1.673	1.695	1.763	1.762	1.762

Table S4 Models selected by NNLS from the data produced by the various methods, including CRYSOL 3.2 with dummy waters, and their percentages, for the SASDM77/AF-Q9UKA9 system. The WAXSiS-based NNLS fits included the two $P(r)$ datasets and either one of the CRYSOL version-based dataset (e.g., "w CR 2.8") or both CRYSOL versions-based datasets ("all").

Model #	$P(r)$ no err wt	$P(r)$ err wt	CRYSOL 2.8	CRYSOL 3.2	WAXSiS w CR 2.8	WAXSiS w CR 3.2	WAXSiS all
0							
20			14.2	23.9	14.1	10.3	13.3
460			19.4				
2020	37.8	23.0					
2030				7.1			
2610		8.3					
3330							
3430		6.7			2.0	2.9	3.5
6680	19.3	23.0	24.3	18.0	23.9	21.8	24.3
7190				0.4			
7290			1.3				
7320			0.4		2.3		1.7
7560	0.5				2.2		
8150	3.7					9.8	
8550	0.1						
8560	1.0			7.8	7.8	1.2	2.0
8570				1.5			
10330		10.4					
10900			7.9	12.8	5.0		
11920	13.0						
12140				1.5			
12300	1.0	20.1	2.8				
12570	3.3						
12700				0.5		11.1	10.9
13440	2.6						
14320	0.9				1.6	5.5	5.1
15210	16.6						
15240				0.2			
15250	0.2	8.5	22.1	13.2	32.5	29.4	32.3
16620			1.2	13.2	8.5	7.9	6.7
16930			6.4				0.3
Sum %	100.0	100.0	100.0	100.0	100.0	100.0	100.0
χ^2	1.279	1.493	1.208	1.240	1.179	1.176	1.176

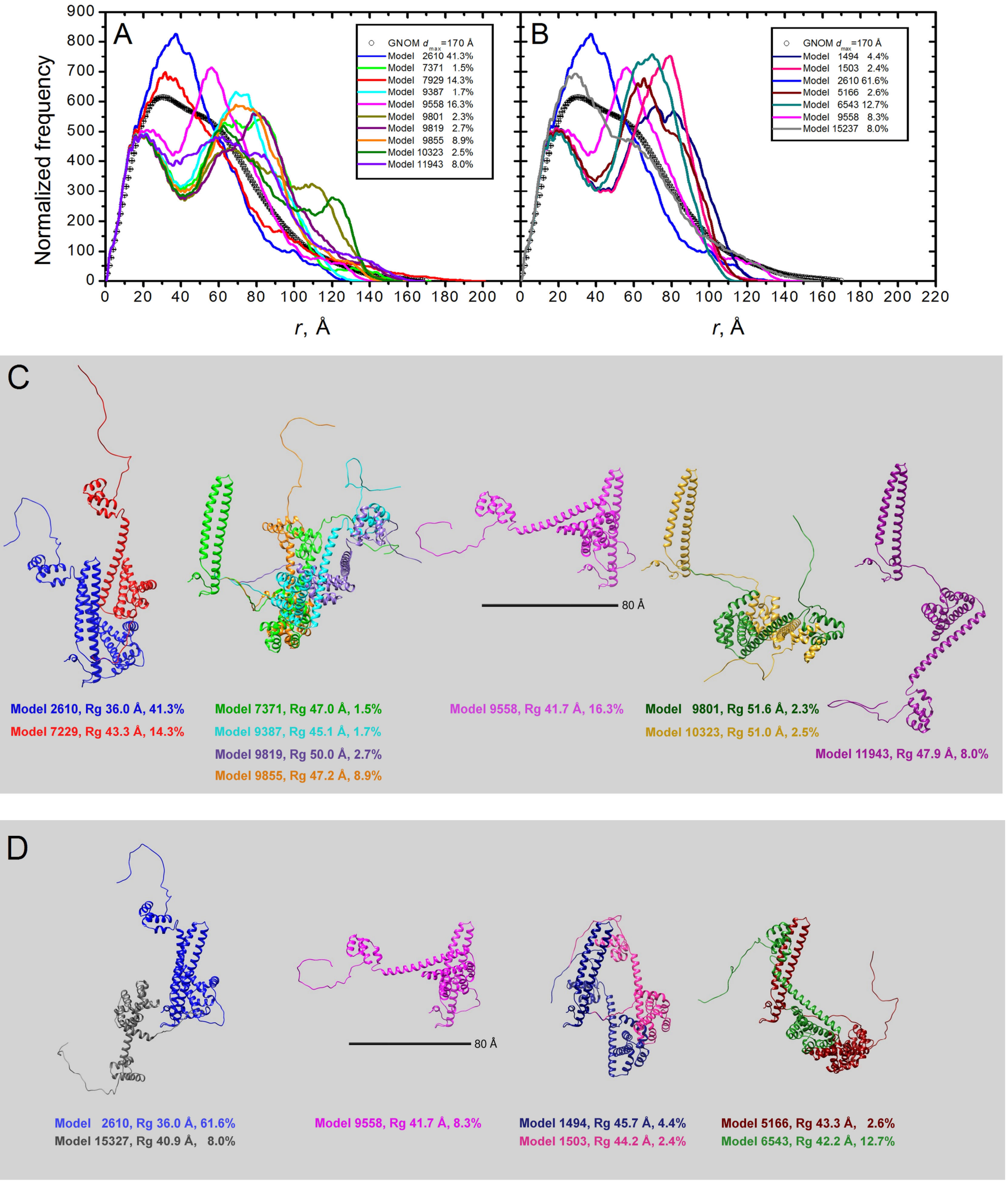

Figure S1 **A,B** $P(r)$ of the individual MMC-generated Q16543 models selected by NNLS without (**A**) and with (**B**) error weighting, with their percent contribution (various colours, see inside panels), overlaid to the experimentally-derived $P(r)$ for SASDBP9. Model numbering is that of the original MMC pool. **C,D** Ribbon representations of the models selected by NNLS without (**C**) or with (**D**) error weighting. The models were all superposed using the N-terminal 25-110 sequence, and they were grouped into 5 (**C**) or 4 (**D**) classes according to a rough estimation of their respective $P(r)$ shapes as plotted in **A** and **B**. Together with their percent contribution, the R_g of each model is also reported.

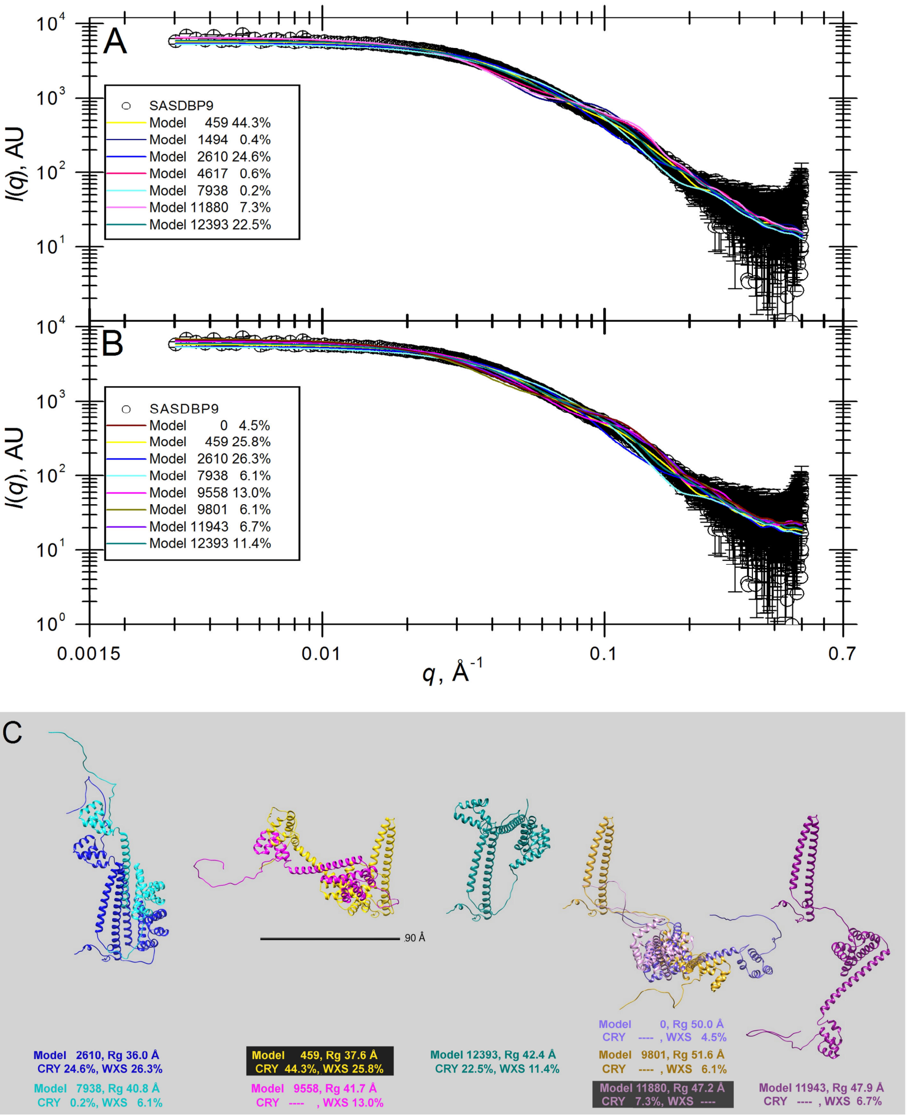

Figure S2 A,B $I(q)$ of the individual MMC-generated Q16543 models selected by NNLS from the CRYSOL 2.8-produced set (**A**), and from the WAXSiS-produced set comprising only the models selected by both the two $P(r)$ and the $I(q)$ CRYSOL 2.8 NNLS fits (**B**), with their percent contribution (various colours, see inside panels), superposed to the SASDBP9 experimental data. Model numbering is that of the original MMC pool. **C** Ribbon representations of the models selected by NNLS in panels **A** and **B**. The models were all superposed using the N-terminal 25-110 sequence, and they were grouped using the $P(r)$-derived clustering (**Figure S1C**) into four classes with one additional class (model 12393). Together with their percent contribution in each NNLS fit (CRY, CRYSOL; WXS, WAXSiS), the R_g of each model is also reported.

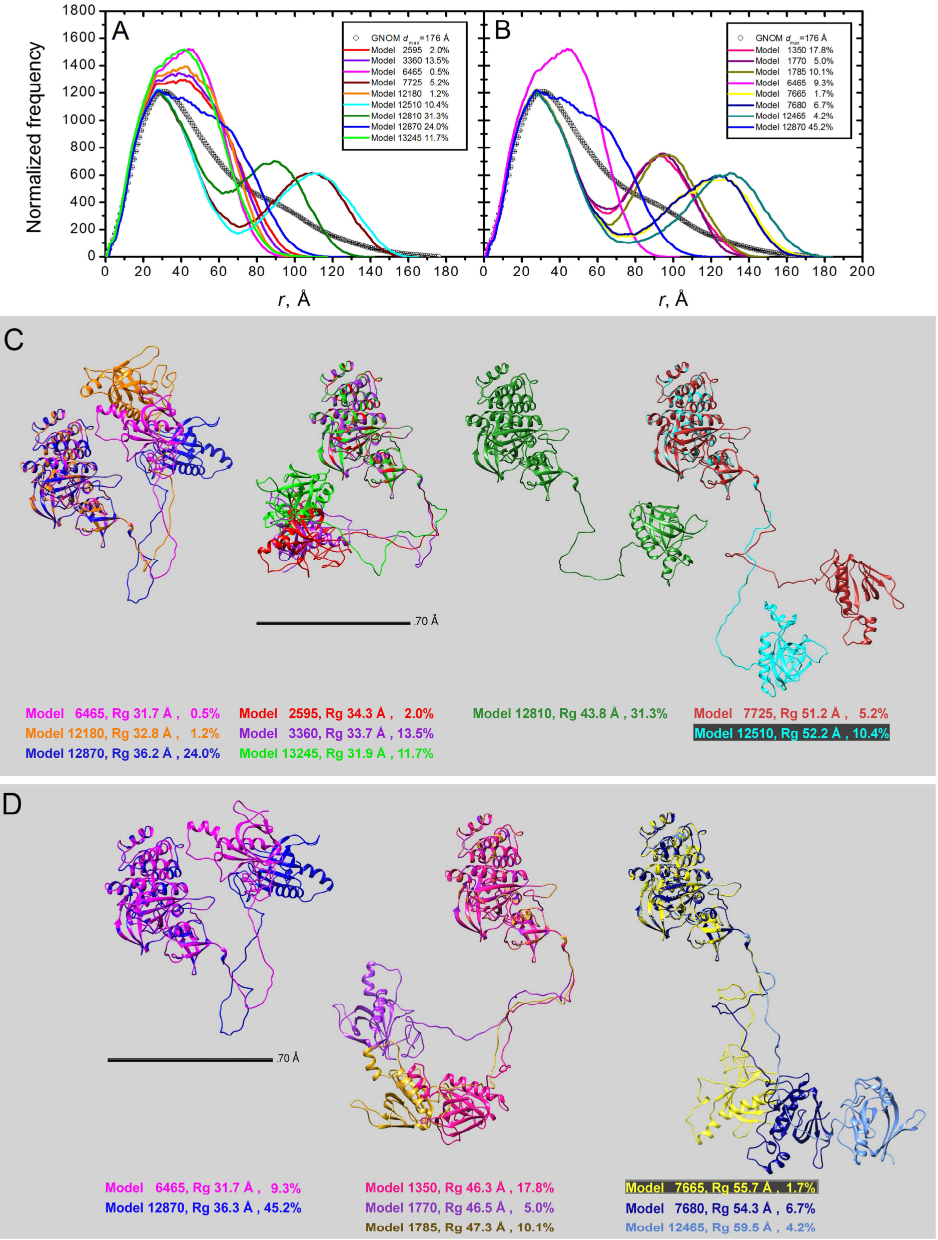

Figure S3 **A,B** $P(r)$ of the individual MMC-generated Q06187 models selected by NNLS without (**A**) and with (**B**) error weighting, with their percent contribution (various colours, see inside panels), overlaid to the experimentally-derived $P(r)$ for SASDF83. Model numbering is that of the original MMC pool. **C,D** Ribbon representations of the models selected by NNLS without (**C**) or with (**D**) error weighting. The models were all superposed using the C-terminal 218-659 sequence, and they were grouped into four (**C**) or three (**D**) classes according to a rough estimation of their respective $P(r)$ shapes as plotted in **A** and **B**, with an additional separation based on the spatial location of the N-terminal domain (left two classes in panel **C**). Together with their percent contribution, the R_g of each model is also reported.

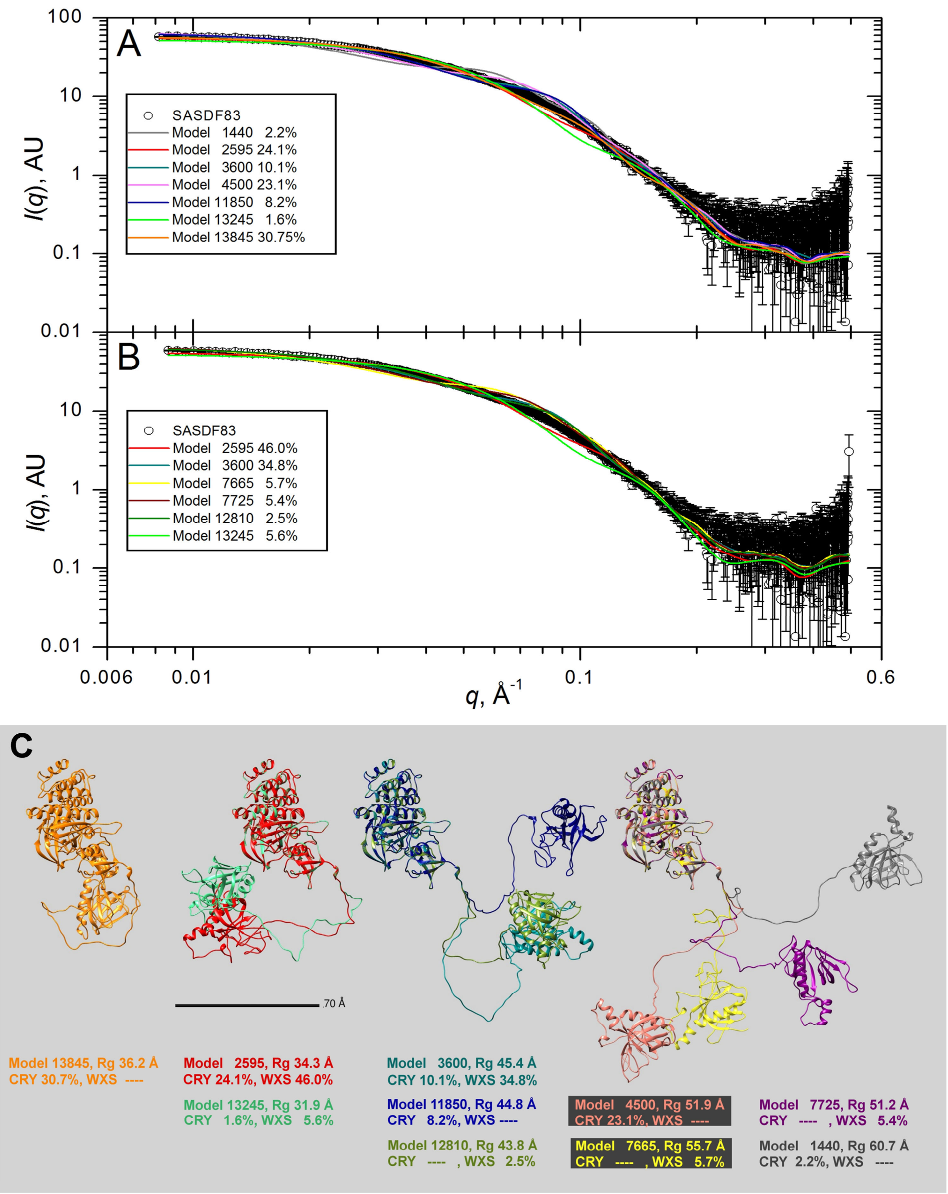

Figure S4 **A,B** $I(q)$ vs. q of the individual MMC-generated Q06187 models selected by NNLS from the CRYSOL 2.8-produced set (**A**), and from the WAXSiS-produced set comprising only the models selected by both the two $P(r)$ and the $I(q)$ CRYSOL 2.8 NNLS fits (**B**), with their percent contribution (various colours, see inside panels), superposed to the SASDF83 experimental data. Model numbering is that of the original MMC pool. **C,D** Ribbon representations of the models selected by NNLS in panels **A** and **B**, respectively. The models were all superposed using the C-terminal 218-659 sequence, and they were grouped using the $P(r)$-derived clustering of **Figure S3C**, with the replacement of the leftmost class with a single model. Together with their percent contribution in each NNLS fit (CRY, CRYSOL; WXS, WAXSiS), the R_g of each model is also reported.

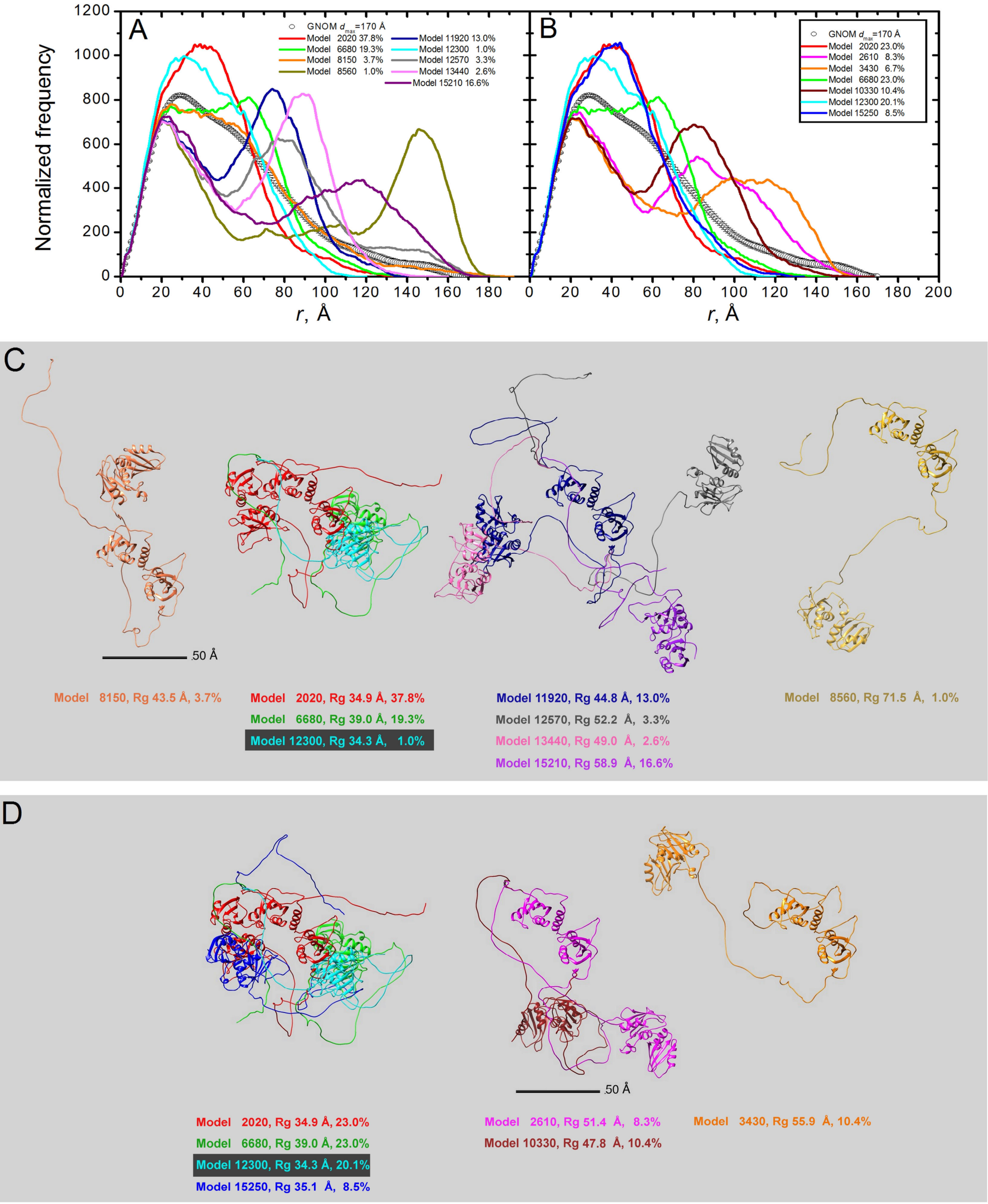

Figure S5 **A,B** $P(r)$ of the individual MMC-generated Q9UKA9 models selected by NNLS without (**A**) and with (**B**) error weighting, with their percent contribution (various colours, see inside panels), overlaid to the experimentally-derived $P(r)$ for SASDM77. Model numbering is that of the original MMC pool. **C,D** Ribbon representations of the models selected by NNLS without (**C**) or with (**D**) error weighting. The models were all superimposed using the N-terminal 63-270 domain, and they were grouped into four (**C**) or three (**D**) classes starting from their respective $P(r)$ shapes as plotted in **A** and **B**, also considering the R_g values, shown together with the models' percent contribution.

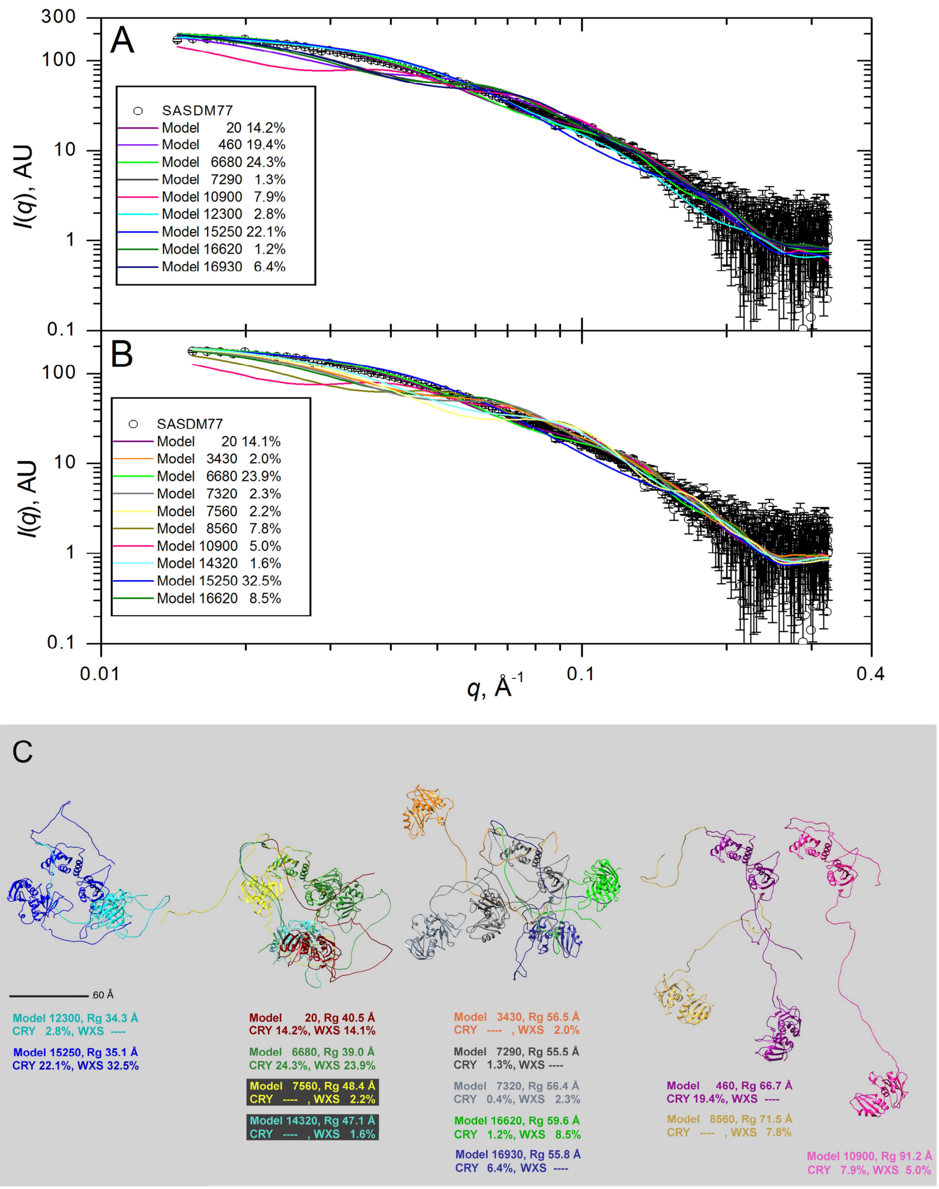

Figure S6 **A,B** *I*(*q*) vs. *q* of the individual MMC-generated Q9UKA9 models selected by NNLS from the CRYSOL 2.8-produced set (**A**), and from the WAXSiS-produced set comprising only the models selected by both the two *P*(*r*) and the *I*(*q*) CRYSOL 2.8 NNLS fits (**B**), with their percent contribution (various colours, see inside panels), superimposed to the SASDM77 experimental data. Model numbering is that of the original MMC pool. **C,D** Ribbon representations of the models selected by NNLS in panels **A** and **B**, respectively. The models were all superimposed using the N-terminal 63-270 domain, and they were grouped using the *P*(*r*)-derived clustering of **Figure S3C**, with the removal of the leftmost class and the insertion of a single very extended model. Together with their percent contribution in each NNLS fit (CRY, CRYSOL; WXS, WAXSiS), the R_g of each model is also reported.
